# Identification of a Novel QTL for Panicle Length From Wild Rice (*Oryza minuta*) by Specific Locus Amplified Fragment Sequencing and High Density Genetic Mapping

**DOI:** 10.3389/fpls.2018.01492

**Published:** 2018-10-16

**Authors:** Zhengzheng Zhu, Xiaoqiong Li, Yu Wei, Sibin Guo, Aihua Sha

**Affiliations:** ^1^Hubei Collaborative Innovation Center for Grain Industry, Yangtze University, Jingzhou, China; ^2^Guangxi Key Laboratory of Rice Genetics and Breeding, Rice Research Institute, Guangxi Academy of Agricultural Science, Nanning, China

**Keywords:** introgression lines, yield related traits, RILs, SNPs, linkage map

## Abstract

Wild rice possesses a large number of valuable genes that have been lost or do not exist in cultivated rice. To exploit the desirable gene controlling panicle length (PL) in wild rice *Oryza minuta*, a recombinant inbred line (RIL) population was constructed that was derived from a cross between the long panicle introgression line K1561 with *Oryza minuta* segments and a short panicle accession G1025. Specific Locus Amplified Fragment (SLAF) sequencing technology was used to uncover single nucleotide polymorphisms (SNPs) and construct the high-density genetic linkage map. Using 201 RIL populations, a high-density genetic map was developed, and spanned 2781.76 cM with an average genetic distance 0.45 cM. The genetic map was composed of 5, 521 markers on 12 chromosomes. Based on this high-density genome map, quantitative trait loci (QTL) for PL were analyzed for 2 years under four environments. Seven QTLs were detected, which were distributed within chromosomes 4, 9, and 10, respectively. *pl4.1* was detected twice, and *pl10.1* was only detected once. Although *pl9.1* was only detected once, it was very near *pl9.2* in the genetic map which was detected three times. Thus, we speculate one major QTL exists in the region of *pl9.1* and *pl9.2* to control PL (temporarily referred to as *pl9*). *pl9* is a potentially novel allele derived from *Oryza minuta*, and it can be used for genetic improvement of cultivar rice.

## Introduction

Rice is the most widely consumed staple food for a large part of the world’s human population, especially in Asia. To meet consumption demand of the growing world population, high-yield variety breeding is one of the major targets for modern breeders. In China, rice yield has been greatly improved during past decades owing to breeding of dwarfism, utilization of hybrid vigor, and cultivation and extension of super rice varieties. However, the narrow genetic basis of the super rice varieties has currently limited further improvements of yield. These yield improvements will likely only be achieved by expanding the available genetic resources.

Cultivated rice (*Oryza sativa* L.) is domesticated from wild rice, which has 22 species that are classified into 10 distinct types according to their genomes. Wild rice species contain many genes that are extremely valuable for genetic improvements of cultivated rice (Guo et al., 2009). Sourcing and using favorable wild rice genes may be an effective way to overcome the yield plateaus in cultivated rice.

*Oryza minuta* (*O. minuta*) is a tetraploid wild relative of cultivated rice, and possesses many resistance and yield improvement related source genes (Rahman et al., 2007). Rahman et al. (2007) developed F_2:3_ families by crossing the introgression line (IL) consisting of at least 14 segments of *O. minuta* with a Korean *japonica* rice variety, and performed QTL mapping for 16 agronomic traits. Of the 22 novel yield-related QTLs detected, 57% were derived from *O. minuta.*

In previous work, we constructed a set of ILs by distant hybridization and backcross, using IR24 (*O. sativa L*) as the recipient and *O. minuta* as the donor; this was combined with embryo rescue and molecular marker assisted selection. QTLs for 12 agronomic traits such as grain weight, grain number, panicle length, panicle number etcetera were mapped using this set of ILs. A total of 28 QTLs for yield related traits were identified, of which, 46.4% of notable QTLs were from *O. minuta* (Guo, 2009).

Rice yield is determined by panicles per plant, spikelets per panicle, seed setting rate and grain weight (Xing and Zhang, 2010). Panicle length (PL) correlates with rice yield, and thus, can be used as criteria for yield breeding (Zhang et al., 2015). A number of QTLs for PL have been mapped, and are located on almost all of the 12 rice chromosomes (Guo and Hong, 2010; Liu et al., 2011; Zhang et al., 2015; Bai et al., 2016; Sun et al., 2017). Some genes/QTLs responsible for PL regulation have been cloned. Most of them regulate the growth of primary or second branches and spikelets by affecting meristematic activity; these include *Ghd7*, *Ghd7.1*, *Short Panicle 1*, *Dense and Erect Panicle1*, *Dense and Erect Panicle2* and *OsRAMOSA2* (Xue et al., 2008; Huang et al., 2009; Li S. et al., 2009, Li et al., 2010; Yan et al., 2013; Lu et al., 2017). Some genes control PL by regulating cell wall components and nutrients necessary for growth; examples of these are *Dense and Erect Panicle3*, *OsCD1*, and *OsARG* (Qiao et al., 2011; Luan et al., 2011; Ma et al., 2013). Other genes regulate PL by modulating hormone metabolism, such as *LP*, *OsPIN5b*, and *OsGRF4* (Li et al., 2011; Lu et al., 2015; Sun et al., 2016). In addition, *LON GPANICLE1* (*LP1*) encodes a protein of unknown function containing a Remorin_C domain (Liu et al., 2016). Two single nucleotide polymorphisms (SNPs) of *LP1* lead to amino acid changes that affect PL.

In this study, we performed QTL analysis for PL using a recombinant inbred line (RIL) population derived from the cross of G1025 and K1561. K1561 is one of a set of 192 ILs that were developed from backcross progenies (BC_4_F_2_) derived from a cross between IR24 and *O. minuta* (Guo, 2009), which shows excellent agronomic traits such as long panicles and high 1000-grain weight. G1025 is an excellent restorer line which is widely used in Guangxi Province of China, and has dense grains but short panicles. Seven QTLs were detected, and the region including *pl9.1* and *pl9.2* was considered as one major candidate (temporarily referred as to *pl9*) to affect PL. The *pl9* allele for increasing PL originated from *O. minuta* and it is an excellent candidate for further application to the genetic improvement of cultivated rice.

## Materials and Methods

### Plant Materials and Field Trials

The parental lines G1025 and K1561 along with the 201 RILs were planted in 2014 in Nanning (NN), China in both the early season (February to July) and the late season (July to November), and only in the early season in 2015. In addition, they were planted in 2015 in Wuhan (WH), China from May to October. K1561 is a long panicle variety developed from the backcross progenies of IR24 and *O. minuta* (Guo, 2009), and G1025 is a short panicle restorer line widely used in Guangxi Province of China. Parents and RILs were planted in single plant and each with three lines. The field managements were conducted with regularly cultural methods. Ten main panicles were harvested to measure PL with ruler after mature. The mean values of ten plants with two replicates were used as input data to identify PL QTLs (**Supplementary Table S1**).

### DNA Preparation

The DNA from two parental lines and RILs was extracted following the CTAB procedure (Murray and Thompson, 1980) and was treated with RNase. DNA quality was determined by electrophoretically resolving on a 1.0% agarose gel, and the concentrations were measured with a NanoDrop^TM^ spectrophotometer.

### SLAF Sample Preparation and Sequencing

Fresh leaves were harvested from a single plant of the parents and RILs, and genomic DNA was isolated for analysis using specific-locus amplified fragment sequencing (SLAF-seq) (Sun et al., 2013). Different combinations of restriction enzyme were tested using *in silico* digestion prediction to obtain expected SLAF tags per genome with even distribution in unique genomic regions. Two restriction enzymes (*Hae* III and *Hpy* 166II) were chosen for their uniform distribution and prevalence among simulations of fragment alignments to the reference genome of Nipponbare (*Oryza sativa L. japonica*)^1^. After digestion, different length fragments of genomic DNA were simulated *in silico*. *Arabidopsis thaliana* (ecotype Columbia) was used as the control genome^2^ to verify the restriction digest protocol for accuracy using SOAP software (Li R. et al., 2009).

Restriction digests were performed with 10 μg of genomic DNA from each parent line and RIL, and were followed by ligation reactions which included the addition of A to the 3′end and ligation with the Dual-index adapter. PCR was performed with the diluted restriction-ligation samples, and the PCR products were then purified with a Quick Spin column (Qiagen, Hilden, Germany) and electrophoretically resolved on a 2% (w/v) agarose gel. Fragments with correct lengths were isolated with a Gel Extraction Kit (Qiagen) and prepared for sequencing. Fragments of 264–314 bp were isolated to be used as SLAF tags. The fragments were sequenced with the Illumina HiSeq 2500 system (Illumina, Inc., San Diego, CA, United States) by Biomarker Technologies Corporation (Beijing, China).

### SLAF Sequence Comparison, Polymorphic Analysis, and Identification of the Associated Markers

The SLAF-seq data were processed as per Sun et al. (2013). In brief, poor reads with a quality score < Q30 (<99.9% confidence) were filtered out. The pair-end reads from SLAF-seq were clustered according to sequence similarity, and the reads could be inferred using BLAT with one to one alignment (-tileSize = 10, -stepSize = 5). Identical reads were merged, and reads with > 90% similarity were placed into one SLAF locus (Sun et al., 2013). Alleles were defined in each SLAF locus with minor allele frequency (MAF) evaluation.

In order to ensure a good quality genetic map, SLAFs with a sequence depth of under 10 × parents, below 30% complete degrees, serious partial separation (*p* < 0.05), and heterozygous loci of the two parents were removed. Polymorphic SLAFs with more than three SNPs were removed. A SLAF-tag with the same genotype as the female (G1025) or male (K1561) parent was designated as a SLAF marker and used for the subsequent association analysis. Diploid species have up to 4 base types, and a polymorphic locus between the parents will have a minimum of 2 base types. Those polymorphic SLAF markers were assorted into eight segregation patterns as following: ab × cd, ef × eg, hk × hk, lm × ll, nn × np, aa × bb, ab × cc, and cc × ab. A heterozygous locus with different base types in both parents is indicated with ab × cd. ef × eg indicates that the locus is heterozygous in both parents, but the parents share one identical base. hk × hk indicates that the locus is heterozygous and the base type is the same in both parents. lm × ll indicates that the locus is heterozygous in the female parent but homozygous in the male parent, and the parents share one identical base. nn × np indicates that the locus is homozygous in the female parent but heterozygous in the male parent. aa × bb indicates that the locus is homozygous in both parents but the parent base type is inconsistent, and the parents share one identical base. ab × cc indicates that the locus is heterozygous in the female parent but homozygous in the male parent, and the parent base type is inconsistent. cc × ab indicates that the locus is heterozygous in the male parent but homozygous in the female parent, and the parent base type is inconsistent. The segregation patterns of aa × bb were used for map construction because the RIL mapping population were derived from two homozygous parents with a genotype of aa or bb.

### Linkage Map Construction and QTL Analysis

JoinMap4.0 software (Van Ooijen, 2006) was used to perform linkage analysis with all the genotype data from the RIL mapping population. The SLAFs can be divided into 12 linkage groups (LGs) with positions of the reference genome. The MLOD value between two adjacent markers was determined (Vision et al., 2000), and SLAFs with MLOD values less than 5 were filtered out. Marker HighMap software (Liu et al., 2014) was used to analyze the linear array of markers in each LG, and the genetic distances between pairs of adjacent markers were estimated. QTLs analysis was conducted using the Internal Mapping method with R/qtl software, and 95% Bayes credible interval method (Sen and Churchill, 2001) was used to calculate the confidence intervals of QTLs. The LOD score threshold for significance was determined using 1,000 permutations with R/qtl.

## Results

### SLAF-Seq Data and SLAF Markers

A total of 302.01 Mbp of raw data were generated by DNA high-throughput sequencing. The average of Q30 ratios was 91.92%, and the average of guanine-cytosine (GC) contents was 42.15%. There were 9,353,528 reads from the female parent, 9,125,218 reads from the male parent, and the average number of reads from the RIL population was 1,410,603.

**FIGURE 1:**
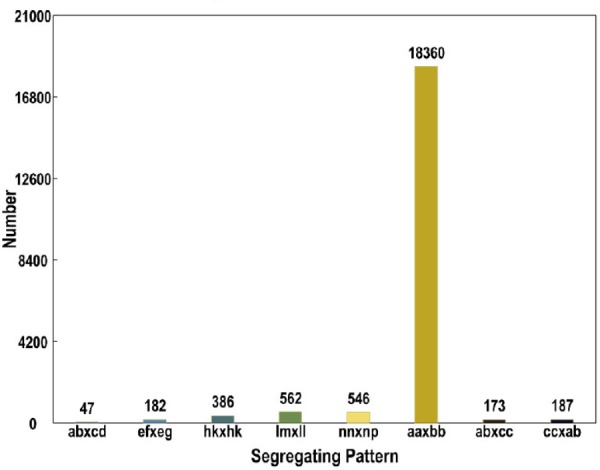
Genotype distribution of SLAF markers. The Y axis represents the number of SLAFs, the X axis represents the type of SLAFs.

The numbers of SLAFs in the male and female parents were 112,450 and 110,284, respectively, correspondingly, the male and female parents had average sequencing depths of 36.02- and 35.48-fold, respectively. The average number of SLAFs was 108,127 in the RIL population, and the coverage was an average of 5.95-fold (**Table 1**). Of the 130,000 high-quality SLAFs detected, 21,549 (or 16.58%) were polymorphic (**Table 2**). A total of 20,443 of the 21,549 polymorphic SLAFs were classified into eight segregation patterns (**Figure 1**). The RIL population was derived from the cross of two parents with an aa or bb homozygous genotype; thus, only the RIL plants with an aa × bb segregation pattern were used for high-density genetic linkage map construction, and a total of 18,360 markers were of this type. Of these 18,360 markers, the high-density genetic map was constructed with 5,960 markers which were homozygous in the two parents and had sequence depth of more than 10-fold with over 70% integrity of SLAF tags.

**Table 1:** Summary of marker depth.

Sample ID	SLAF number	Total depth	Average depth
G1025	112,450	4,050,703	36.02
K1561	110,284	3,912,556	35.48
Average of RILs	108,127	644,993	5.95

**Table 2:** Number of specific length amplified fragment (SLAF) markers on each chromosome.

Chr ID	SALF number	Polymorphic SLAF
Chr01	14,754	1,866
Chr02	12,930	2,052
Chr03	13,889	2,041
Chr04	10,704	1,830
Chr05	10,439	1,627
Chr06	10,544	1,275
Chr07	10,195	2,234
Chr08	8,439	1,770
Chr09	7,025	1,119
Chr10	6,621	1,300
Chr11	6,821	1,181
Chr12	6,983	1,176
Scaffold	10,656	2,078
Total	130,000	21,549

### Genetic Map Construction and Its Basis Information

After linkage analysis, 5,521 of the 5,960 SLAF markers were mapped on the genetic map. On average, these markers had 53.32-fold sequence depths in G1025 (the female parent), 53.20-fold in K1561 (the male parent), and 8.65-fold in each RIL line. The final genetic map included 5,521 markers on the twelve linkage genetic map (LGs) (**Table 3** and **Supplementary Figures S1**, **S2**), and was 2,187.16 cM in length with an inter-marker distance of 0.45 cM (**Table 3**). The largest LG was LG1 with 803 markers, a length of 195.52 cM, and an average distance of 0.24 cM between adjacent markers. The smallest LG was LG8 which only has 211 markers, a length of 156.64 cM and an average distance of 0.78 cM. The largest gap on this map was 14.50 cM, which is located in LG4.

**Table 3:** Basic characteristics of twelve linkage groups.

Linkage group ID	Total marker	Total distance (cM)	Average distance (cM)	Largest gap (cM)
Chr01	803	195.52	0.24	7.91
Chr02	634	182.2	0.29	7.06
Chr03	506	185.71	0.37	7.90
Chr04	350	187.96	0.54	14.50
Chr05	525	184.77	0.35	13.35
Chr06	369	170.33	0.46	10.07
Chr07	225	174.22	0.78	9.76
Chr08	211	156.64	0.75	9.49
Chr09	432	176.93	0.41	8.56
Chr10	521	194.78	0.37	2.35
Chr11	468	192.81	0.41	5.94
Chr12	477	185.29	0.39	12.86
Total	5,521	2,187.16	0.45	109.75

### Evaluation of the Genetic Map

The quality of the genetic map was evaluated by drawing heat maps for the 5,521 SLAF markers using pair-wise recombination values. Adjacent markers in the heat maps have low recombination frequencies, which indicate that the constructed linkage map has good accuracy (**Supplementary Figure S3**).

The haplotype map is reflective of genotyping errors that have occurred by crossover events in an advanced population. According to the haplotype map, most of the recombination blocks were defined with under 0.1% heterozygosity, less than 0.1% missing, and an even distribution of markers on each chromosome (**Supplementary Figure S4**).

Evaluation of collinearity between the genetic map and the rice reference genome was performed by mapping SLAF markers to the rice reference genome, and curves between physical distances and genetic distances were evident for most chromosomes except Chr8 (**Supplementary Figure S5**). The high collinearity suggests that the 5,521 SLAF markers were accurately mapped to the 12 chromosomes.

### QTL Analysis Based on the High-Density Genetic Map

**FIGURE 2:**
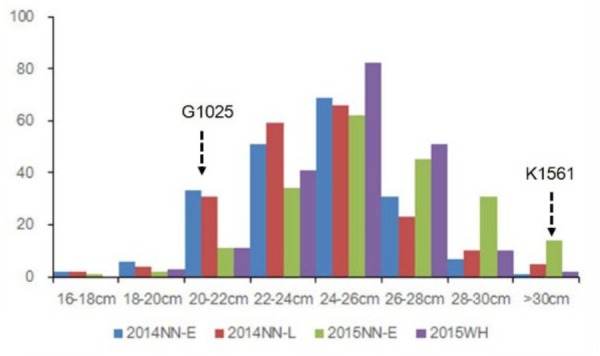
Phenotypic evaluation of PL for G1025, K1561, and RILs. The Y axis represents the number of plant individuals. The X axis is continuous for panicle length: 16 cm < PL ≤ 18 cm; 18 cm < PL ≤ 20 cm; 20 cm < PL ≤ 22 cm; 22 cm < PL ≤ 24 cm; 24 cm < PL ≤ 26 cm; 26 cm < PL ≤ 28 cm; 28 cm < PL ≤ 30 cm; PL > 30 cm.

**FIGURE 3:**
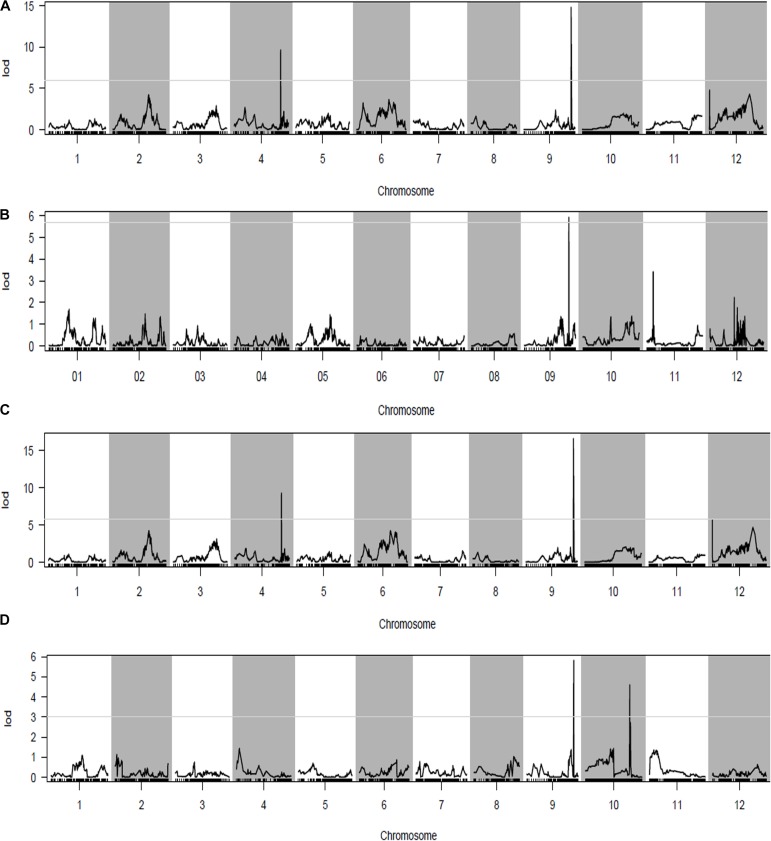
Quantitative trait loci (QTL) analysis with panicle length (PL) using the high-density genetic map. **(A)** QTL analysis of PL in the early season of Nanning (NN) in 2014; **(B)** QTL analysis of PL in the late season of Nanning (NN) in 2014; **(C)** QTL analysis of PL in NN in 2015; **(D)** QTL analysis of PL in Wuhan (WH) in 2015. The horizontal line on the chart represents the LOD threshold.

**FIGURE 4:**
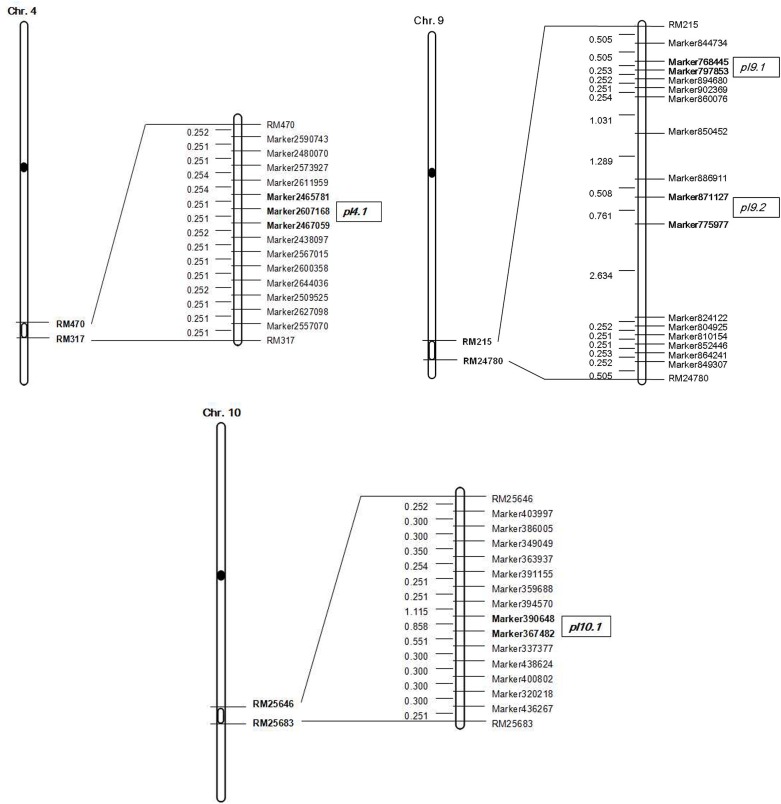
Genetic linkage map with locations of QTLs on chromosomes 4, 9, and 10.

Phenotypic data of the two parents and RILs planted in NN and WH in 2014 and 2015 are presented in **Figure 2** and **Supplementary Table S1**. A total of seven QTLs for PL were detected in RILs (**Table 4** and **Figures 3**, **4**), and mapped to unique positions. The proportion of phenotypic variance explained by a single QTL (*r*2) ranged from 7.67% to 26.99% and LOD scores were from 4.60 to 16.61. The two QTLs located on chromosomes 4 accounted for 14.84% and 14.01% of the phenotypic variation, respectively. Four QTLs were located on chromosome 9, and accounted for 24.46%, 13.63%, 26.99%, and 13.15% of the phenotypic variation, respectively. One QTL was located on chromosome 10, and accounted for 7.67% of phenotypic variation. *pl4.1* was repeatedly detected early in the season in Nanning in 2014 and 2015, and *pl10.1* was only detected in Wuhan in 2015. *pl9.1* was only detected late in the season in season of Nanning, and *pl9.2* could be detected repeatedly early in the season in Nanning in 2014, 2015, and in Wuhan in 2015. Furthermore, *pl9.2* had a larger LOD and it was quite nearby *pl9.1* in the genetic map. We cannot judge whether *pl9.1* and *pl9.2* are the same QTL or two distinguishing QTLs at present, so we temporarily designated *pl9.1* and *pl9.2* as a single major candidate QTL that controls PL (referred as to *PL9*).

**Table 4:** Quantitative trait loci (QTL) analysis of rice panicle length in RILs.

Year	Season^#^	QTL	Chr	Marker interval	Interval (cM)	LOD	Additive effect	r^2^(%)	The donor parent
2014	NN-E	*Pl4.1*	4	Marker2465781-Marker2467059	160.40-160.90	9.66	−1.08	14.84	G1025
		*pl9.2*	9	Marker871127-Marker775977	162.39-163.15	14.81	−1.38	24.46	G1025
2014	NN-L	*pl9.1*	9	Marker768445-Marker797853	158.55-158.80	6.35	−0.93	13.63	G1025
2015	NN-E	*Pl4.1*	4	Marker2465781-Marker2467059	160.40-160.90	9.29	−1.01	14.01	G1025
		*pl9.2*	9	Marker871127-Marker775977	162.39-163.15	16.61	−1.41	26.99	G1025
2015	WH	*pl9.2*	9	Marker871127-Marker775977	162.39-163.15	5.82	−1.48	13.15	G1025
		*Pl10.1*	10	Marker390648-Marker367482	152.74-153.60	4.60	−1.13	7.67	G1025

*^#^NN-E, during the early season in Nanning; NN-L, during the late season in Nanning; WH, in Wuhan.*

**FIGURE 5:**
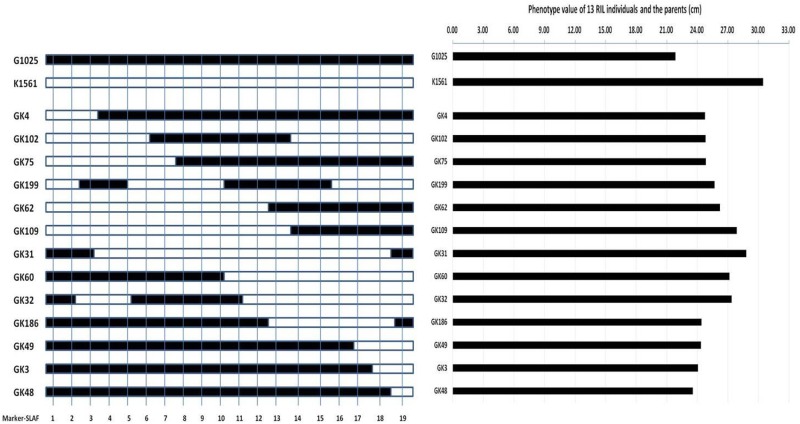
Genotypes and phenotypes of 13 RILs and parents. Left figure: genotypes of 13 RILs and parents; Mark-SLAF1-19, Marker839969, Marker775801, Marker868127, Marker823913, Marker891014, Marker803328, Marker860031, Marker768445, Marker797853, Marker860076, Marker871127, Marker775977, Marker824122, Marker864241, Marker876655, Marker911696, Marker866573, Marker891726, Marker864313, respectively. The solid bar represents segments of G1025, and the hollow bar represents segments of K1561. Right figure: phenotypes of 13 RILs and parents.

In order to evaluate whether the phenotypes were affected by the detected QTLs, we selected 13 out of the 201 RILs to compare their phenotypes with their genotypes. The 13 RILs were homozygous at the loci of *pl4.1*, *pl4.2*, and *pl10* for the genotypes of G1025 (date not shown), but they were homozygous at the loci of *pl9.1* and *pl9.2* for the genotypes of either G1025 or K1561 (**Figure 5**). The PL phenotypes of the 13 RILs were consistent with their respective genotypes (**Table 5** and **Figure 5**).

**Table 5:** Phenotypes of RILs and parents.

RILs or parents	Panicle length (cm)	Phenotype
G1025	21.85	A
K1561	30.45	B
GK4	24.77	A
GK102	24.8	A
GK75	24.83	A
GK199	25.68	B
GK62	26.23	B
GK109	27.87	B
GK31	28.82	B
GK60	27.15	B
GK32	27.36	B
GK186	24.4	A
GK49	24.35	A
GK3	24.07	A
GK48	23.57	A

## Discussion

By combining locus-specific amplification and high-throughput sequencing, SLAF-seq is an effective technology for *de novo* SNP discovery and large-scale genotyping. It has distinguishing characteristics of deep sequencing, but employs a reduced representation strategy, pre-designed reduced representation scheme, and double barcodes. Therefore, it can ensure genotyping accuracy, reduce sequencing costs, reasonably and reliably predict fragmentation efficiency, and allow for the simultaneous genotyping of large populations (Sun et al., 2013). In addition, marker development with SLAF-seq was less time-consuming, less expensive, and more efficient relative to other conventional methods (Zhu et al., 2016).

*O. minuta* possess a large number of desirable source genes which can be used for yield improvement. In a previous study, a set of ILs were constructed by crossing IR24 and *O. minuta*, and an excellent IL, K1561, was selected from this set (Guo, 2009). In order to further exploit and utilize the favorable genes of *O. minuta* in this study, we conducted SLAF-seq to develop SNP markers and to identify QTLs for PL by using the population of RILs. We obtained 302.01 Mbp of reads, including 130,000 high-quality SLAF markers with a polymorphism rate of 14.12%. The obtained markers covered all twelve chromosomes, which had between 1,176 and 2,234 polymorphic SLAF markers on each chromosome. A total of 5,521 high quality polymorphic SLAF markers were used to construct the high-density SNP map. The integrity and accuracy of the markers were much higher, and the quality and quantity of markers met the requirements for construction of a high-density genetic linkage map.

PL is an important yield-related trait that determines the number of spikelets. Based on the high-density genetic linkage map and the phenotypes of G1025, K1561, and the RILs, the seven detected QTLs were distributed on chromosomes 4, 9, and 10. *pl4.1* was repeatedly detected in the early seasons of NN in 2014 and 2015, but was absent in the late seasons of NN in 2014 and WH in 2015. *pl10.1* was only detected in WH in 2015. *pl9.1* was only detected in the late season of NN, but it is very close to *pl9.2* in the genetic map; *pl9.2* was stably detected during the early seasons of NN in 2014 and 2015, and in WH in 2015 with a higher LOD score. The region containing *pl9.1* and *pl9.2* has been temporarily denoted as a major QTL candidate that controls PL (referred as to *PL9*), because we cannot judge whether *pl9.1* and *pl9.2* are the same QTL or two separate QTLs at present. The phenotypes were affected by genotypes at *pl9.1* and *pl9.2* by analysis of 13 RILs, indicating that *Pl9* most likely controls PL.

Up until now, many QTLs for PL have been mapped on nearly all of the twelve chromosomes, however, only a few QTLs were located on chromosome 9. *DEP1* is a pleiotropic QTL responsible for dense panicle, high grain number per panicle and erect panicle, and was defined between the markers RM3700 and RM7424. *DEP1 (LOC_Os09g26999)* has been cloned and encodes a protein containing a phosphatidylethanolamine-binding protein (PEBP)-like domain (Huang et al., 2009). *qPL-9-1* and *qPL-9-2* were mapped between markers RM6570-RM5652 and RM5652-RM410 by using 254 RILs derived from a cross between cultivars Xiushui 79 and C Bao, respectively (Guo and Hong, 2010). The *qPL-9-2* (*LP*) was further narrowed to a 90 kb region of DNA between markers L04 and RM7289, and the candidate gene *LOC_Os09g28300* has been cloned (Liu et al., 2016). *MPL9*, was mapped between markers RM215 and RM1013 by using RILs derived from the cross of Teqing and Minghui 63, but it was detected during only 1 year (Liu et al., 2011). One QTL for PL was mapped between markers S9058.3-RM7175 by using 178 F_7_ RILs from a cross of *japonica* rice line “SNUSG1” and *indica* rice line “Milyang23” (Lim et al., 2014). The mapping or cloning of these QTLs or genes was conducted based on the population derived from the crosses of cultivated varieties.

The mapping population in this study was derived from the cross of a restorer line G1025 and an IL with the segments of *O. minuta* K1561. One major QTL, *pl9*, was defined within 304 Kb between SLAF Markers 768,445 and 775,977. It is a potentially novel allele from *O. minuta*. *pl9* is within the region of rice genome SSR markers RM24780-RM215 (International Rice Genome Sequencing Project, 2005), which is nearby but not the same as *MPl9* (Liu et al., 2011), and it was different with other QTLs or genes that have been identified on the chromosome 9. Other QTLs for PL have been detected in *O. minuta*, and include *pl6*, *pl7*, *pl8* identified on chromosome 6, 7, 8, respectively (Rahman et al., 2007). In addition, QTLs for PL have also been identified in other wild rice species. For example, the chromosome 6 located *spd6* is responsible for small panicles and dwarfness, and was identified in *Oryza rufipogon Griff.* (Shan et al., 2009). Results such as these indicate that potential alleles controlling PL might exist in the genome of wild rice.

## Conclusion

In conclusion, we identified a novel QTL for PL (*pl9*) from *O. minuta* by construction of the high-density SNP map and QTL association analysis. Refined mapping of the *pl9* locus will be conducted to identify the candidate genes. On the other hand, the *pl9* allele from *O. minuta* can also be directly used for genetic improvement of cultivar rice.

## Author Contributions

ZZ extracted DNA, prepared the library, and wrote the manuscript. XL performed SNP data analyses and wrote the manuscript. YW constructed the linkage map. SG and AS designed the experiments and supervised the study.

## Conflict of Interest Statement

The authors declare that the research was conducted in the absence of any commercial or financial relationships that could be construed as a potential conflict of interest.

## Footnotes

1 http://rice.plantbiology.msu.edu/

2 http://www.ncbi.nlm.nih.gov/genome/?term=Arabidopsis%20thaliana

## Supplementary Material

FIGURE S1 High-density genetic map constructed by 5521 SLAF Markers.

FIGURE S2 Distribution of Markers on chromosomes that were applied to genetic map construction.

FIGURE S3 Heat map of the high-density genetic map. Each cell represents the recombination rate of two markers. Yellow, red, and purple represent the recombination rate ranging from lower to higher.

FIGURE S4 Haplotype map of the genetic map. Blue represents K1561, red represents G1025, green indicates heterozygous type, and gray represents deletions.

FIGURE S5 The collinearity of 12 chromosomes with the rice reference genome. The x-axis indicates the genetic distance of rice chromosomes, and the y-axis represents the linearity order of the physical position in the rice genome. All 5521 SLAF markers in these chromosomes are plotted as dots on the Figure. Different colors indicate different chromosomes.

TABLE S1 Panicle length data of two parents and RILs.

## References

[B1] Bai X. Zhao H. Huang Y. Xie W. Han Z. Zhang B. (2016). Genome-wide association analysis reveals different genetic control in panicle architecture between Indica and Japonica rice. *Plant Genome* 9 1–10. 10.3835/plantgenome2015.11.0115 27898816

[B2] Guo S. (2009). *Development and Characterization of Substation Lines from a Cross of Oryza sativa and Oryza minuta.* Dissertation thesis, Huzhong Agricultural University, Wuhan.

[B3] Guo S. Qin F. Zhang D. Lin X. (2009). Characterization of interspecific hybrids and backcross progenies from a cross between *Oryza minuta* and *Oryza sativa*. *Sci. China Ser. C Life Sci.* 52 1148–1155. 10.1007/s11427-008-0155-0 20016972

[B4] Guo Y. Hong D. (2010). Novel pleiotropic loci controlling panicle architecture across environments in japonica rice (*Oryza sativa* L.). *J. Genet. Genomics* 37 533–544. 10.1016/S1673-8527(09)60073-4 20816386

[B5] Huang X. Qian Q. Liu Z. Sun H. He S. Luo D. (2009). Natural variation at the DEP1 locus enhances grain yield in rice. *Nat. Genet.* 41 494–497. 10.1038/ng.352 19305410

[B6] International Rice Genome Sequencing Project (2005). The map-based sequence of the rice genome. *Nature* 436 793–800. 10.1038/nature03895 16100779

[B7] Li F. Liu W. Tang J. Chen J. Tong H. Hu B. (2010). Rice dense and erect panicle 2 is essential for determining panicle outgrowth and elongation. *Cell Res.* 20 838–849. 10.1038/cr.2010.69 20502443

[B8] Li M. Tang D. Wang K. Wu X. Lu L. Yu H. (2011). Mutations in the F-box gene larger panicle improve the panicle architecture and enhance the grain yield in rice. *Plant Biotechnol. J.* 9 1002–1013. 10.1111/j.1467-7652.2011.00610.x 21447055

[B9] Li R. Yu C. Li Y. Lam T. W. Yiu S. M. Kristiansen K. (2009). SOAP2: an improved ultrafast tool for short read alignment. *Bioinformatics* 25 1966–1967. 10.1093/bioinformatics/btp336 19497933

[B10] Li S. Qian Q. Fu Z. Zeng D. Meng X. Kyozuka J. (2009). Short panicle1 encodes a putative PTR family transporter and determines rice panicle size. *Plant J.* 58 592–605. 10.1111/j.1365-313X.2009.03799.x 19154200

[B11] Lim J. H. Yang H. J. Jung K. H. Yoo S. C. Paek N. C. (2014). Quantitative trait locus mapping and candidate gene analysis for plant architecture traits using whole genome re-sequencing in rice. *Mol. Cells* 37 149–160. 10.14348/molcells.2014.2336 24599000 PMC3935628

[B12] Liu D. Ma C. Hong W. Huang L. Liu M. Liu H. (2014). Construction and analysis of high-density linkage map using high-throughput sequencing data. *PLoS One* 9:e98855. 10.1371/journal.pone.0098855 24905985 PMC4048240

[B13] Liu E. Liu Y. Wu G. Zeng S. Thi T. Liang L. (2016). Identification of a candidate gene for panicle length in rice (Oryza sativa L.) via association and linkage analysis. *Front. Plant Sci.* 7:596. 10.3389/fpls.2016.00596 27200064 PMC4853638

[B14] Liu T. Li L. Zhang Y. Xu C. Li X. Xing Y. (2011). Comparison of quantitative trait loci for rice yield, panicle length and spikelet density across three connected populations. *J. Genet.* 90 377–382. 10.1007/s12041-011-0083-9 21869494

[B15] Lu G. Coneva V. Casaretto J. Ying S. Mahmood K. Liu F. (2015). OsPIN5b modulates rice (*Oryza sativa*) plant architecture and yield by changing auxin homeostasis, transport and distribution. *Plant J.* 83 913–925. 10.1111/tpj.12939 26213119

[B16] Lu H. Dai Z. Li L. Wang J. Miao X. Shi Z. (2017). OsRAMOSA2 Shapes panicle architecture through regulating pedicel length. *Front. Plant Sci.* 8:1538. 10.3389/fpls.2017.01538 28955349 PMC5601049

[B17] Luan W. Liu Y. Zhang F. Song Y. Wang Z. Peng Y. (2011). OsCD1 encodes a putative member of the cellulose synthase-like D sub-family and is essential for rice plant architecture and growth. *Plant Biotech. J.* 9 513–524. 10.1111/j.1467-7652.2010.00570.x 20955181

[B18] Ma X. Cheng Z. Qin R. Qiu Y. Heng Y. Yang H. (2013). OsARG encodes an arginase that plays critical roles in panicle development and grain production in rice. *Plant J.* 73 190–200. 10.1111/j.1365-313x.2012.05122.x 26011250

[B19] Murray M. G. Thompson W. F. (1980). Rapid isolation of high molecular-weight plant DNA. *Nucleic Acids Res.* 8 4321–4325. 10.1093/nar/8.19.4321 7433111 PMC324241

[B20] Qiao Y. Piao R. Shi J. Lee S. Jiang W. Kim B. (2011). Fine mapping and candidate gene analysis of dense and erect panicle 3, DEP3, which confers high grain yield in rice (*Oryza sativa* L.). *Theor. Appl. Genet.* 122 1439–1449. 10.1007/s00122-011-1543-6 21318372

[B21] Rahman M. Chu S. H. Choi M. S. Choi M. S. Qiao Y. Jiang W. (2007). Identification of QTLs for some agronomic traits in rice using an introgression line from *Oryza minuta*. *Mol. Cells* 24 16–26. 17846495

[B22] Sen S. Churchill G. A. (2001). A statistical framework for quantitative trait mapping. *Genetics* 159 371–387. 11560912 10.1093/genetics/159.1.371 PMC1461799

[B23] Shan J. Zhu M. Shi M. Gao J. Lin H. (2009). Fine mapping and candidate gene analysis of spd6, responsible for small panicle and dwarfness in wild rice (*Oryza rufipogon* Griff.). *Theor. Appl. Genet.* 119 827–836. 10.1007/s00122-009-1092-4 19588119

[B24] Sun P. Zhang W. Wang Y. He Q. Shu F. Liu H. (2016). OsGRF4 controls grain shape, panicle length and seed shattering in rice. *J. Integr. Plant Biol.* 58 836–847. 10.1111/jipb.12473 26936408 PMC5089622

[B25] Sun X. W. Liu D. Y. Zhang X. F. Li W. B. Liu H. Hong W. (2013). SLAF-seq: an efficient method of large-scale De Novo SNP discovery and genotyping using high-throughput sequencing. *PLoS One* 8:e58700. 10.1371/journal.pone.0058700 23527008 PMC3602454

[B26] Sun Z. Yin X. Ding J. Yu D. Hu M. Sun X. (2017). QTL analysis and dissection of panicle components in rice using advanced backcross populations derived from Oryza Sativa cultivars HR1128 and ‘Nipponbare’. *PLoS One* 12:e0175692. 10.1371/journal.pone.0175692 28422981 PMC5396889

[B27] Van Ooijen J. (2006). *VoorripsR: Joinmap4.0. Software for the Calculation of Genetic Linkage Maps in Experimental Populations.* Wageningen: Kyazma BV.

[B28] Vision T. J. Brown D. G. Shmoys D. B. Durrett R. T. Tanksley S. D. (2000). Selective mapping: a strategy for optimizing the construction of high-density linkage maps. *Genetics* 155 407–420. 10790413 10.1093/genetics/155.1.407 PMC1461083

[B29] Xing Y. Zhang Q. (2010). Genetic and molecular bases of rice yield. *Annu. Rev. Plant Biol.* 61 421–442. 10.1146/annurev-arplant-042809-112209 20192739

[B30] Xue W. Xing Y. Weng X. Zhao Y. Tang W. Wang L. (2008). Natural variation in Ghd7 is an important regulator of heading date and yield potential in rice. *Nat. Genet.* 40 761–767. 10.1038/ng.143 18454147

[B31] Yan W. Liu H. Zhou X. Li Q. Zhang J. Lu L. (2013). Natural variation in Ghd7.1 plays an important role in grain yield and adaptation in rice. *Cell Res.* 23 969–971. 10.1038/cr.2013.43 23507971 PMC3698629

[B32] Zhang L. Wang J. Wang J. Wang L. Ma B. Zeng L. (2015). Quantitative trait locus analysis and fine mapping of the qPL6 locus for panicle length in rice. *Theor. Appl. Genet.* 128 1151–1161. 10.1007/s00122-015-2496-y 25821195

[B33] Zhu W. Huang L. Chen L. Yang J. Wu J. Qu M. (2016). A high-density genetic linkage map for cucumber (*Cucumis sativus* L.): based on Specific Length Amplified Fragment (SLAF) sequencing and QTL analysis of fruit traits in cucumber. *Front. Plant Sci.* 7:437. 10.3389/fpls.2016.00437 27148281 PMC4835494

