# Supplementary figures and images for: Identification of a Novel QTL for Panicle Length From Wild Rice (Oryza minuta) by Specific Locus Amplified Fragment Sequencing and High Density Genetic Mapping
Source: Front Plant Sci. 2018 Oct 16;9:1492. doi: 10.3389/fpls.2018.01492 (PMC6232755; doi:10.3389/fpls.2018.01492)

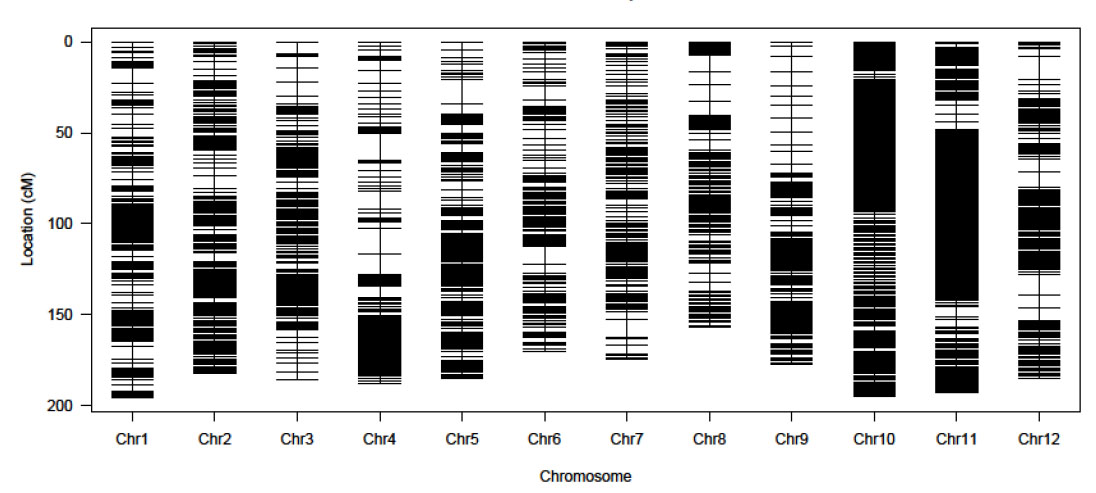

Supplement: FIGURE S1 — High-density genetic map constructed by 5521 SLAF Markers. [file Image_1.JPEG]

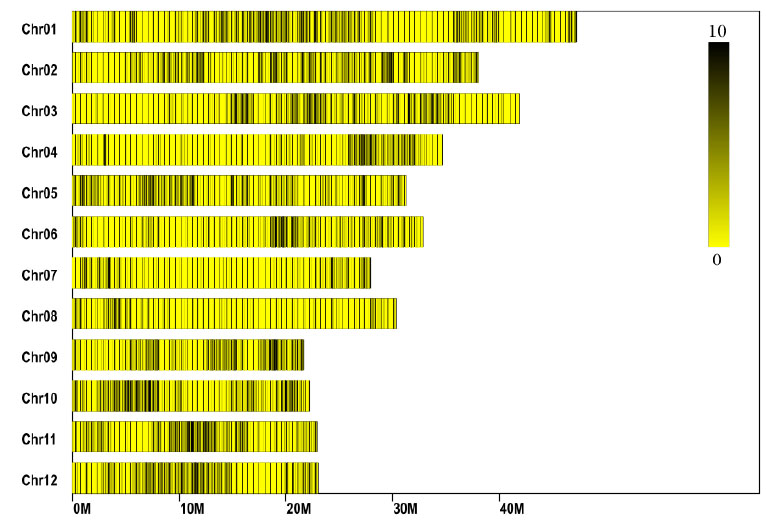

Supplement: FIGURE S2 — Distribution of Markers on chromosomes that were applied to genetic map construction. [file Image_2.JPEG]

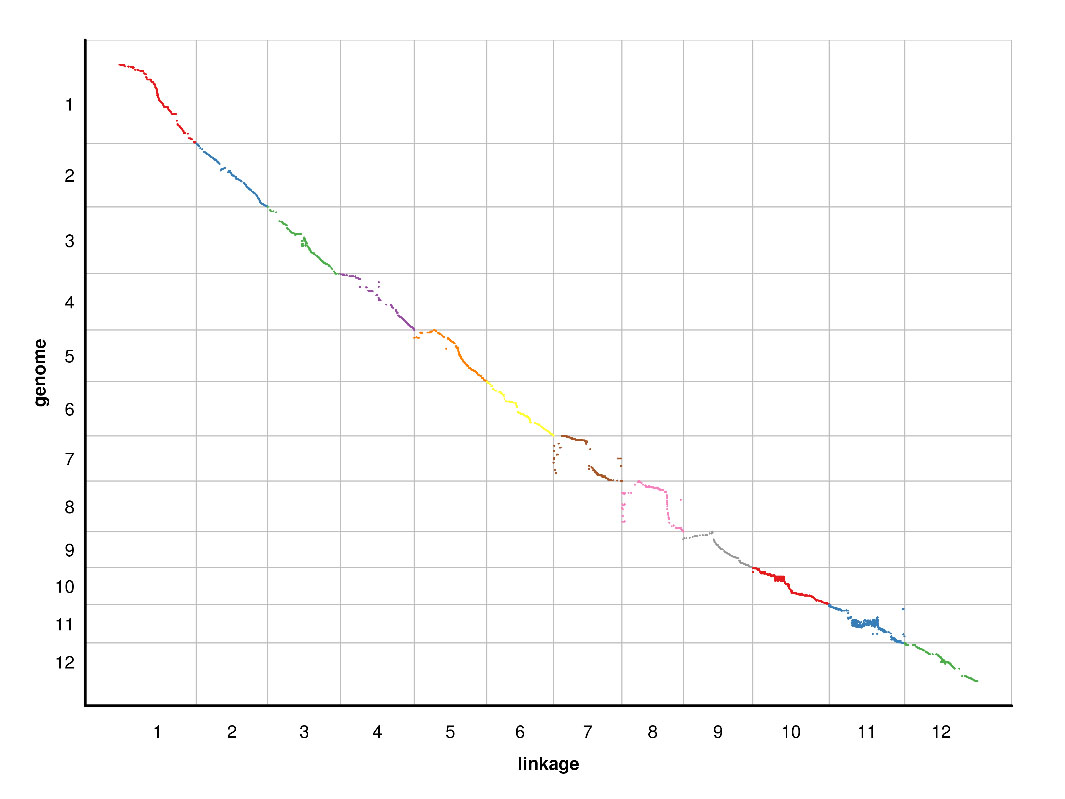

Supplement: FIGURE S3 — Heat map of the high-density genetic map. Each cell represents the recombination rate of two markers. Yellow, red, and purple represent the recombination rate ranging from lower to higher. [file Image_3.JPEG]

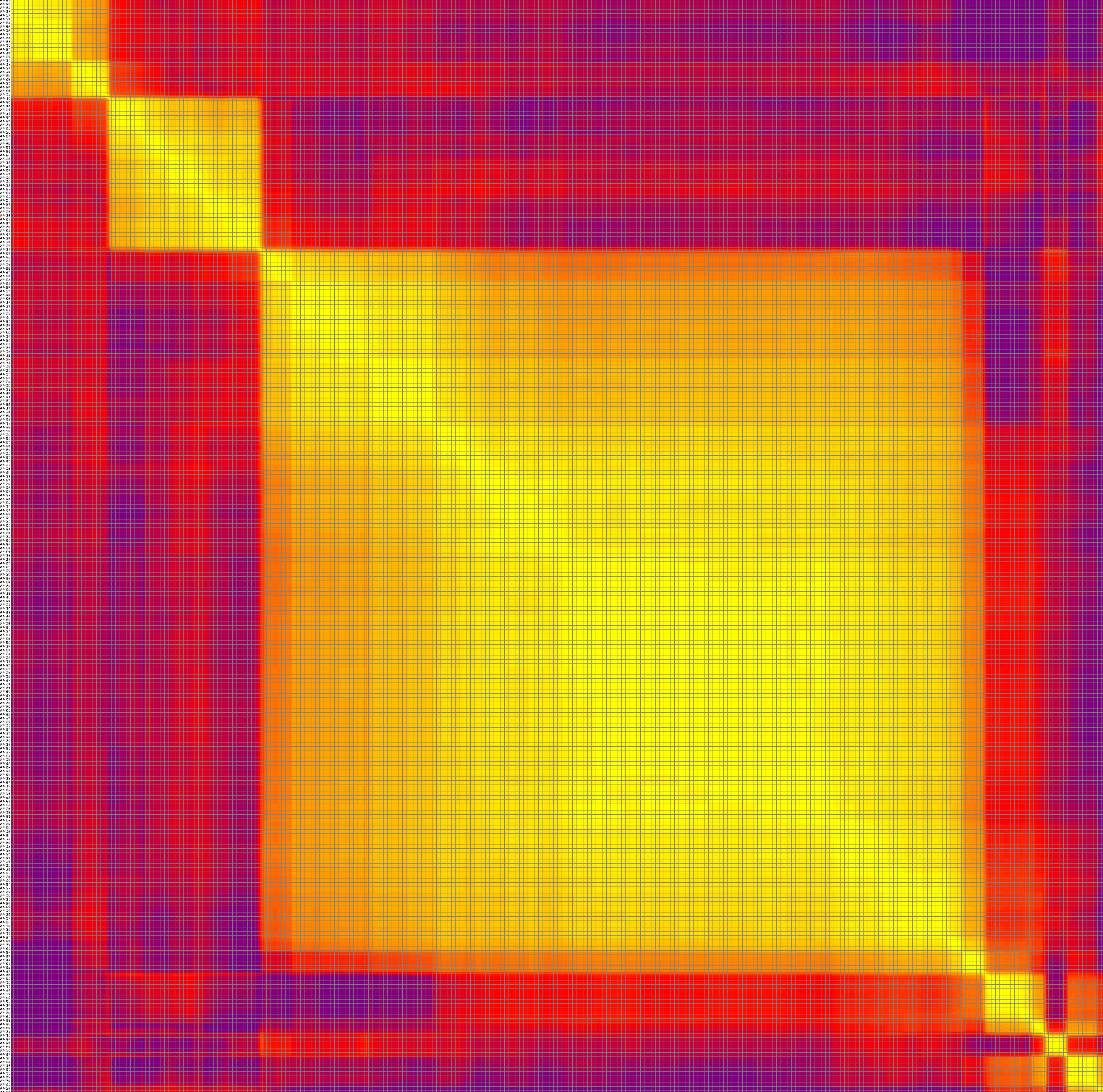

Supplement: FIGURE S4 — Haplotype map of the genetic map. Blue represents K1561, red represents G1025, green indicates heterozygous type, and gray represents deletions. [file Presentation_1.ZIP › Supplementary Figure S3/rice.Chr01.heatMap.png]

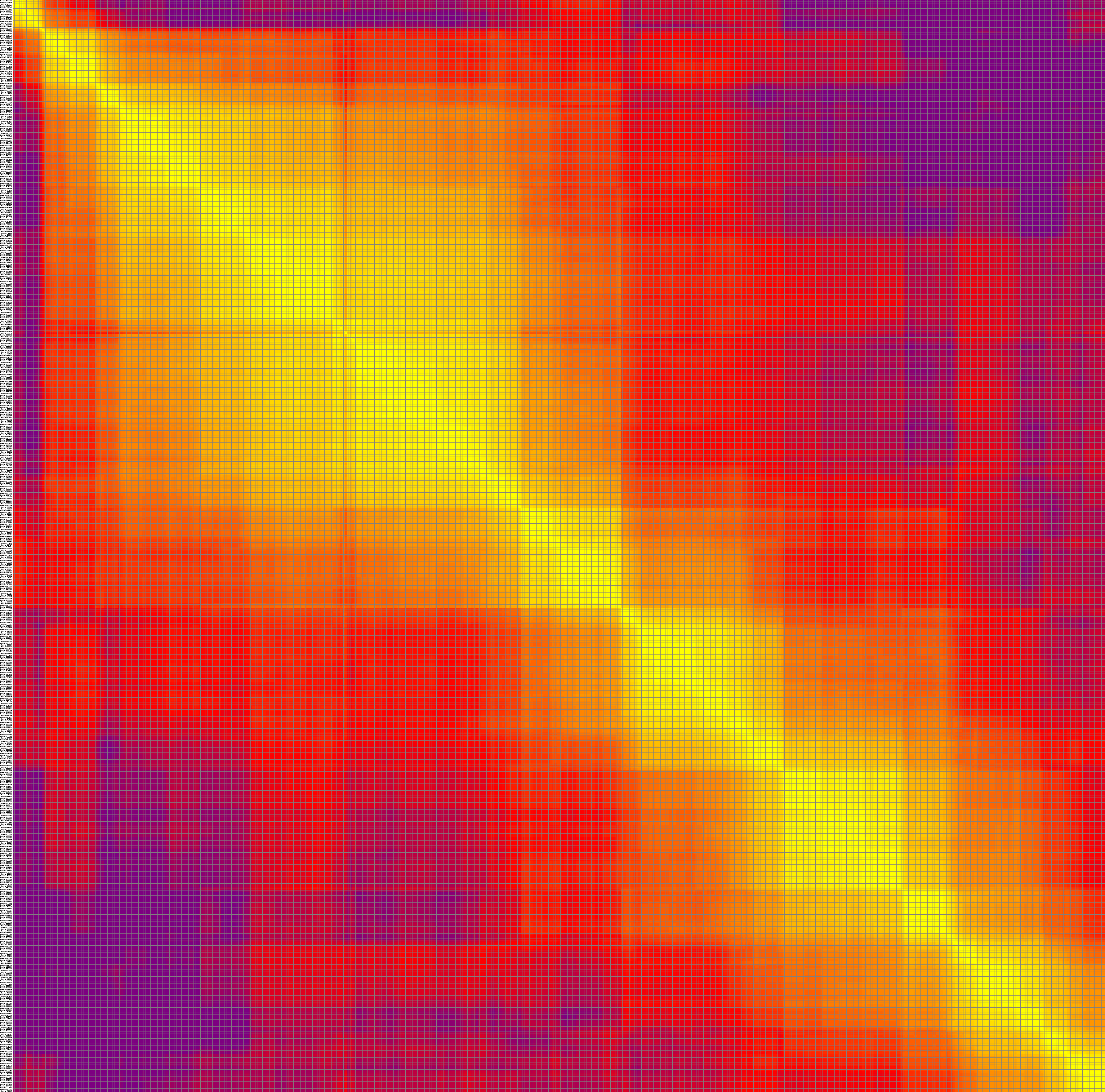

Supplement: FIGURE S4 — Haplotype map of the genetic map. Blue represents K1561, red represents G1025, green indicates heterozygous type, and gray represents deletions. [file Presentation_1.ZIP › Supplementary Figure S3/rice.Chr02.heatMap.png]

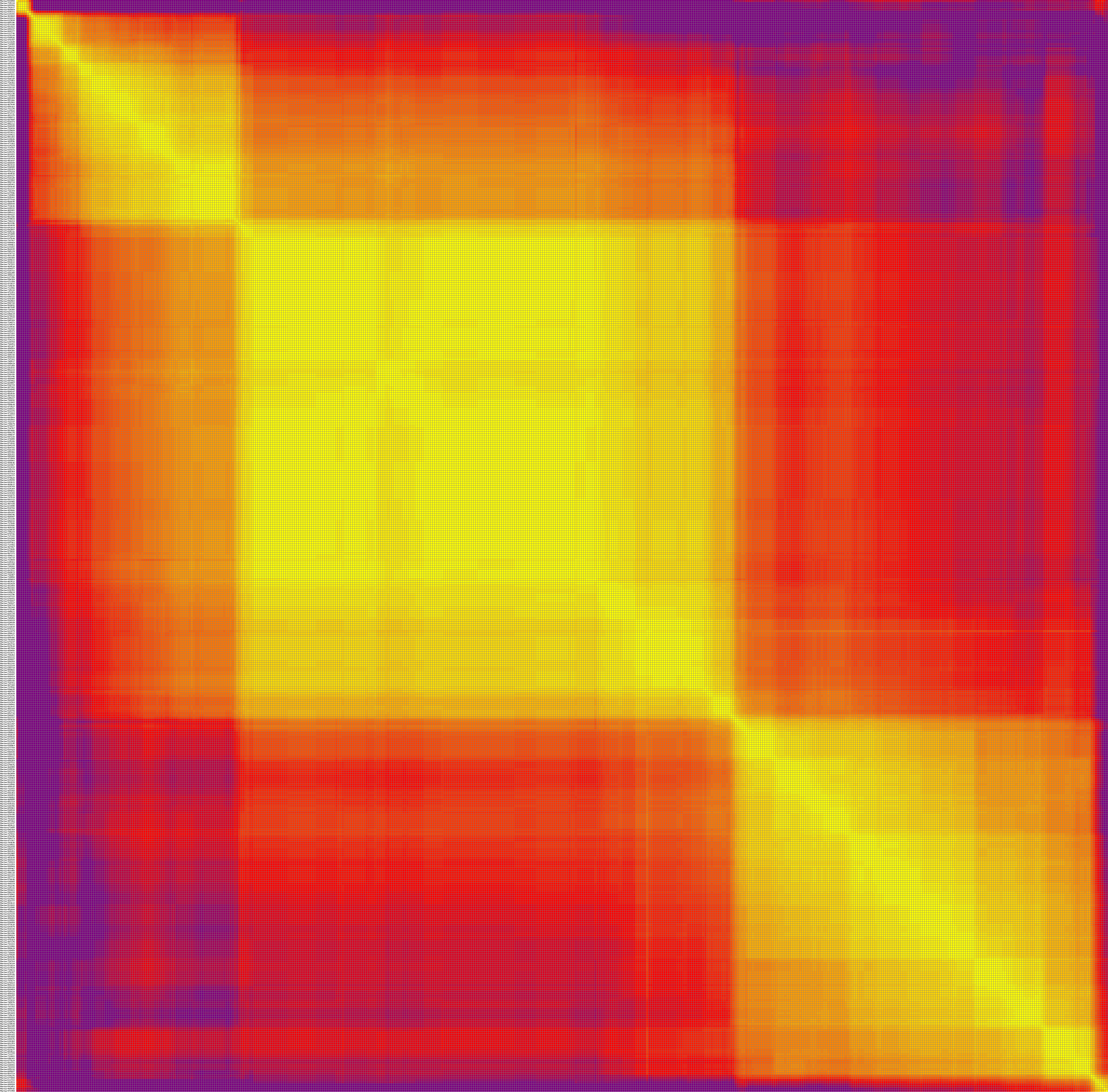

Supplement: FIGURE S4 — Haplotype map of the genetic map. Blue represents K1561, red represents G1025, green indicates heterozygous type, and gray represents deletions. [file Presentation_1.ZIP › Supplementary Figure S3/rice.Chr03.heatMap.png]

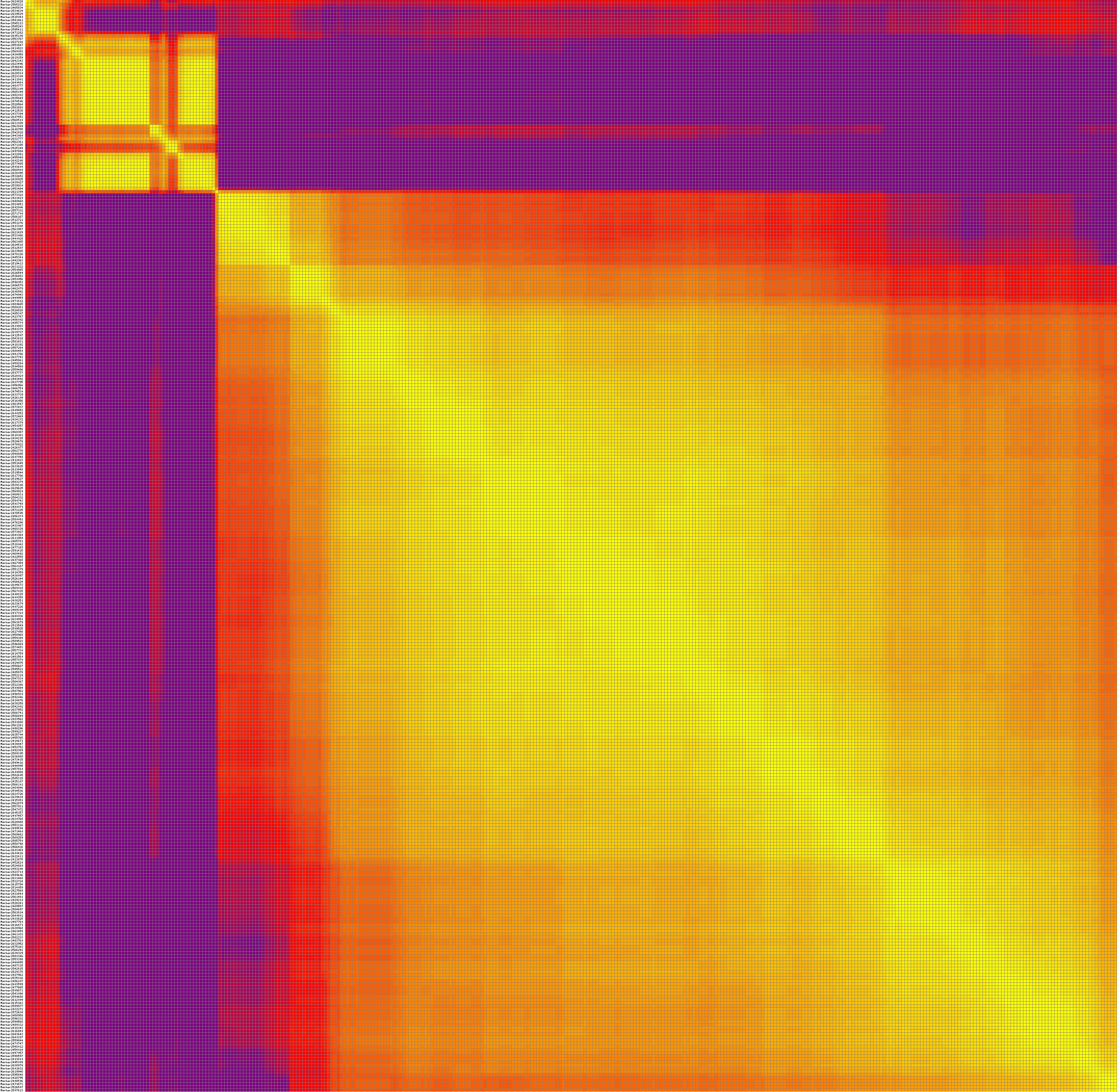

Supplement: FIGURE S4 — Haplotype map of the genetic map. Blue represents K1561, red represents G1025, green indicates heterozygous type, and gray represents deletions. [file Presentation_1.ZIP › Supplementary Figure S3/rice.Chr04.heatMap.png]

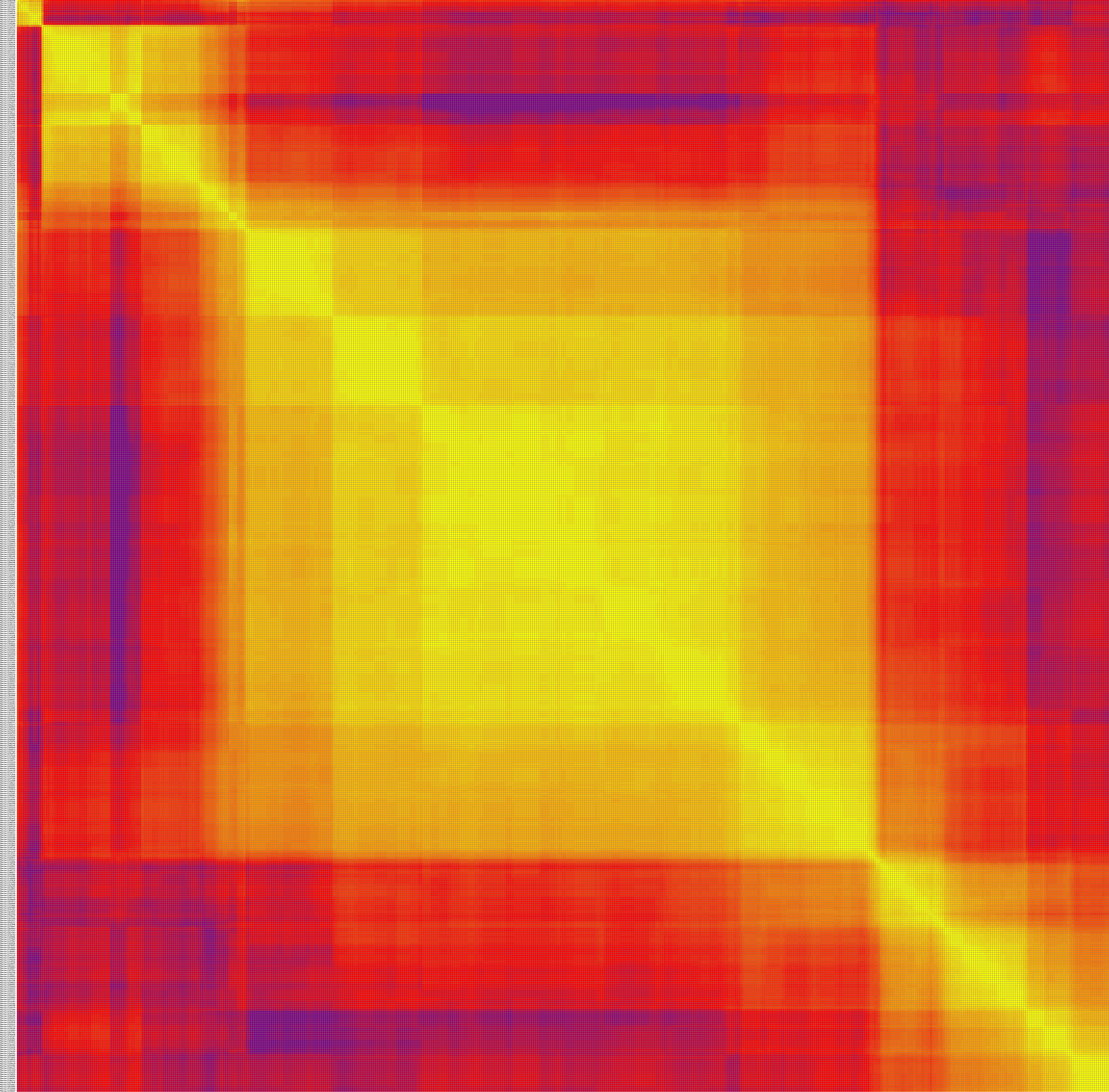

Supplement: FIGURE S4 — Haplotype map of the genetic map. Blue represents K1561, red represents G1025, green indicates heterozygous type, and gray represents deletions. [file Presentation_1.ZIP › Supplementary Figure S3/rice.Chr05.heatMap.png]

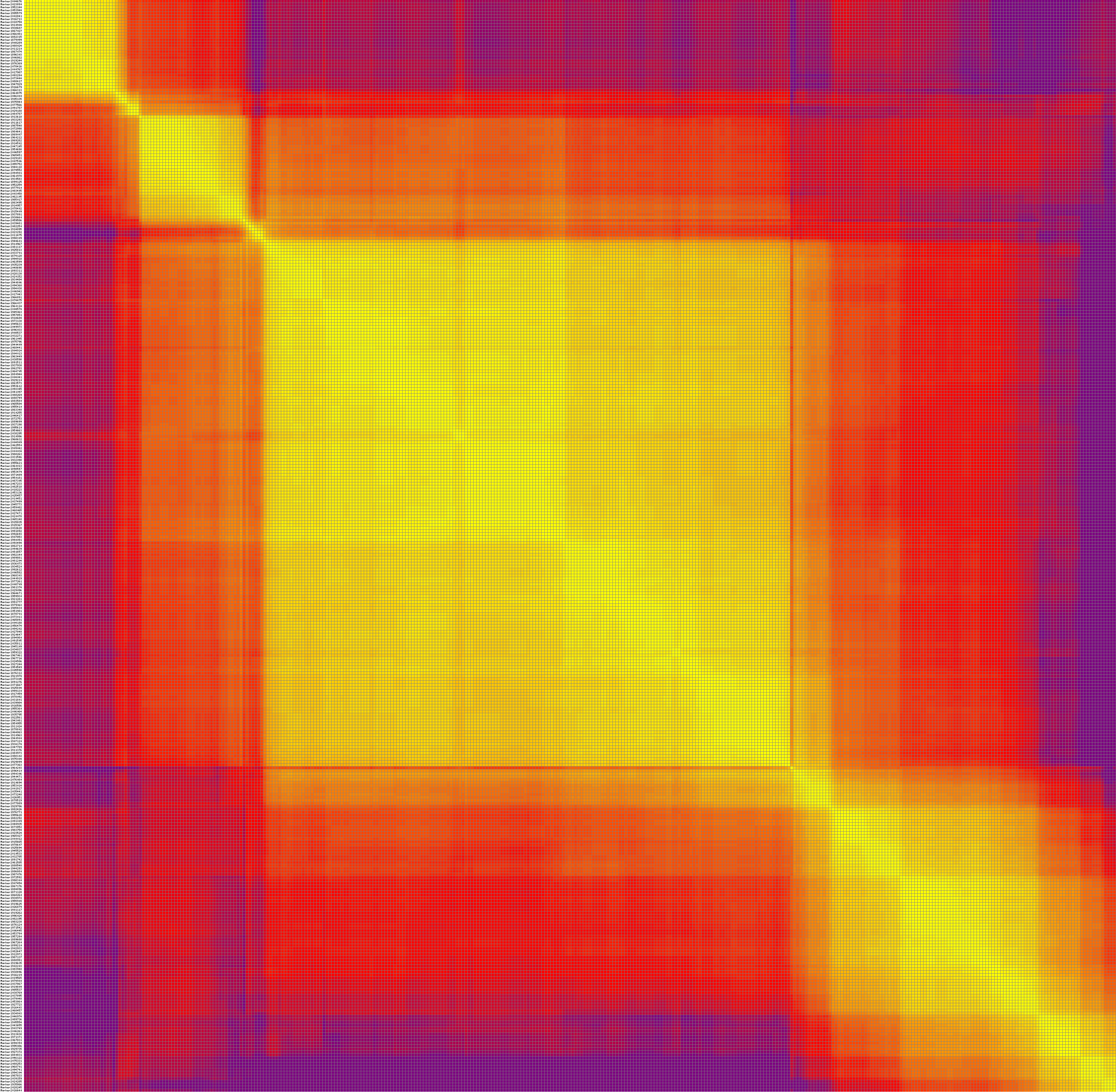

Supplement: FIGURE S4 — Haplotype map of the genetic map. Blue represents K1561, red represents G1025, green indicates heterozygous type, and gray represents deletions. [file Presentation_1.ZIP › Supplementary Figure S3/rice.Chr06.heatMap.png]

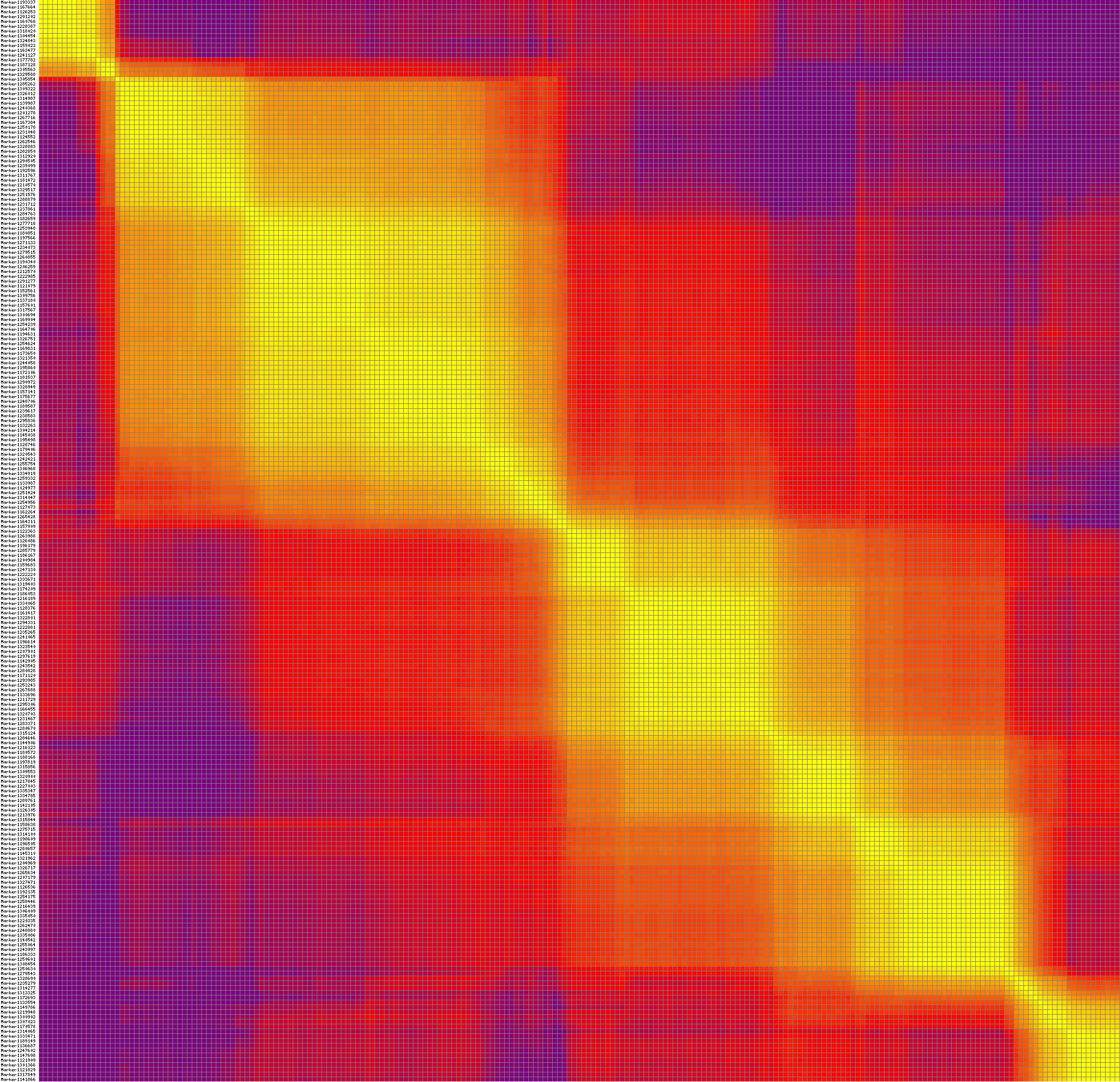

Supplement: FIGURE S4 — Haplotype map of the genetic map. Blue represents K1561, red represents G1025, green indicates heterozygous type, and gray represents deletions. [file Presentation_1.ZIP › Supplementary Figure S3/rice.Chr07.heatMap.png]

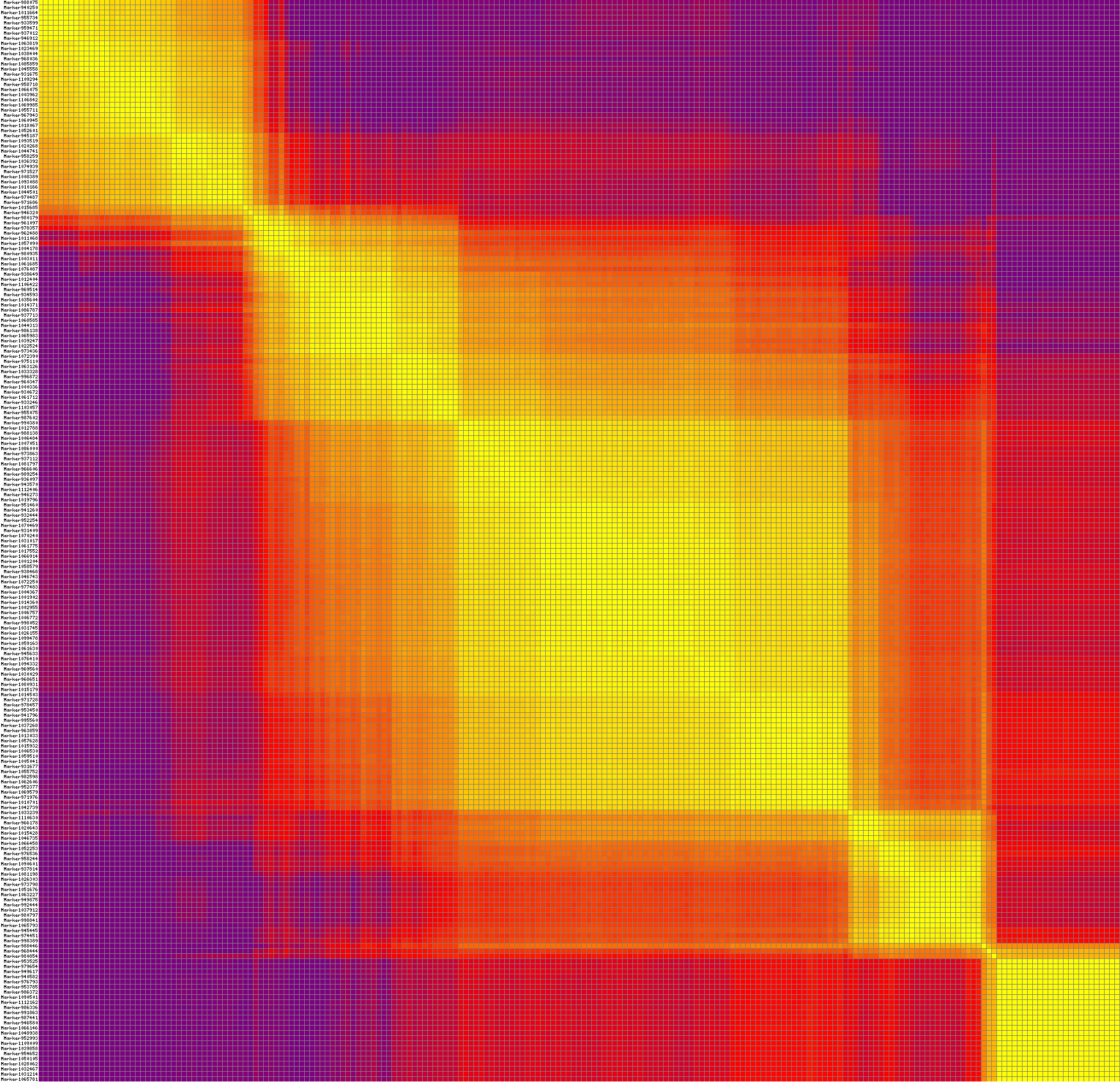

Supplement: FIGURE S4 — Haplotype map of the genetic map. Blue represents K1561, red represents G1025, green indicates heterozygous type, and gray represents deletions. [file Presentation_1.ZIP › Supplementary Figure S3/rice.Chr08.heatMap.png]

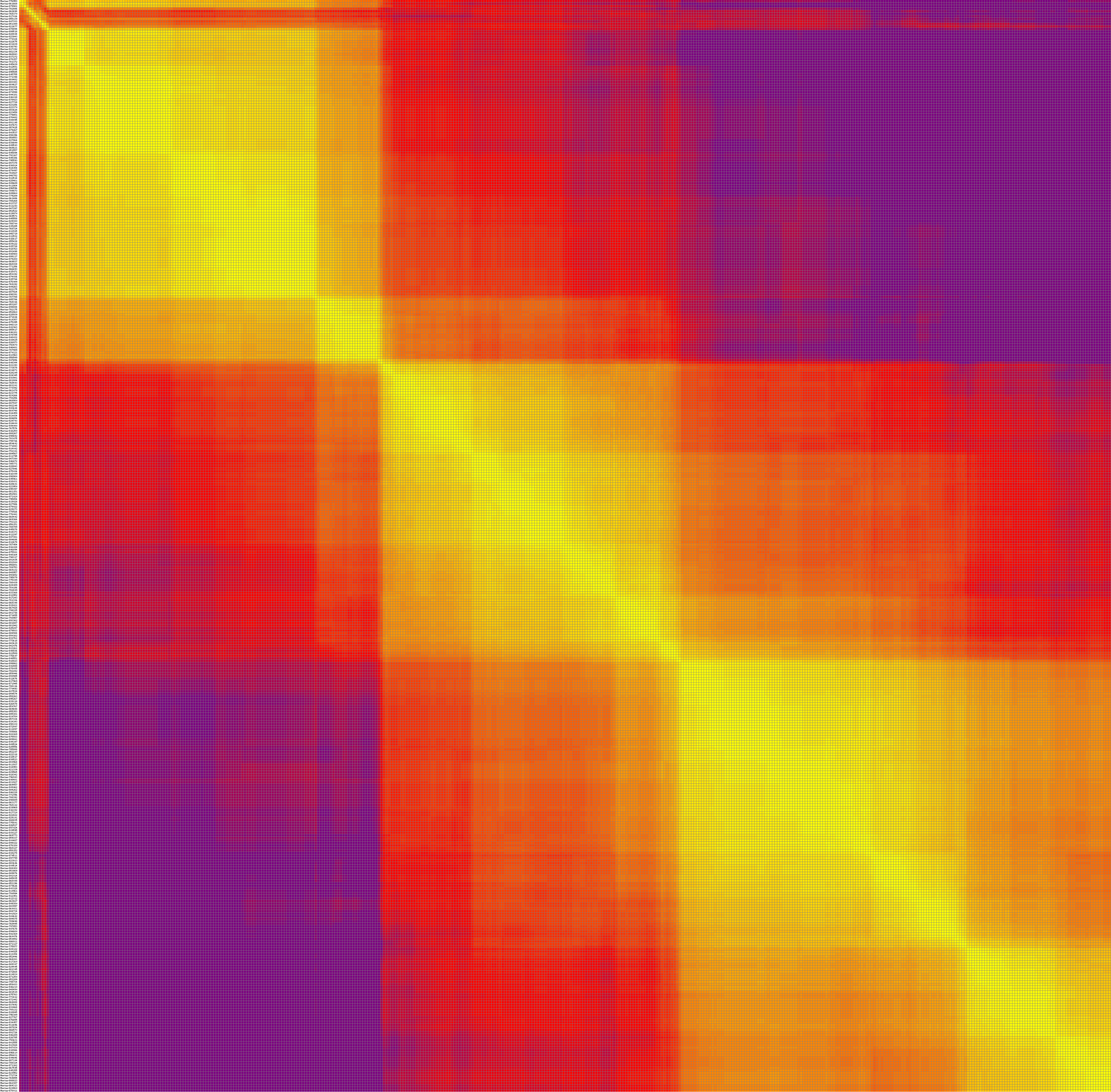

Supplement: FIGURE S4 — Haplotype map of the genetic map. Blue represents K1561, red represents G1025, green indicates heterozygous type, and gray represents deletions. [file Presentation_1.ZIP › Supplementary Figure S3/rice.Chr09.heatMap.png]

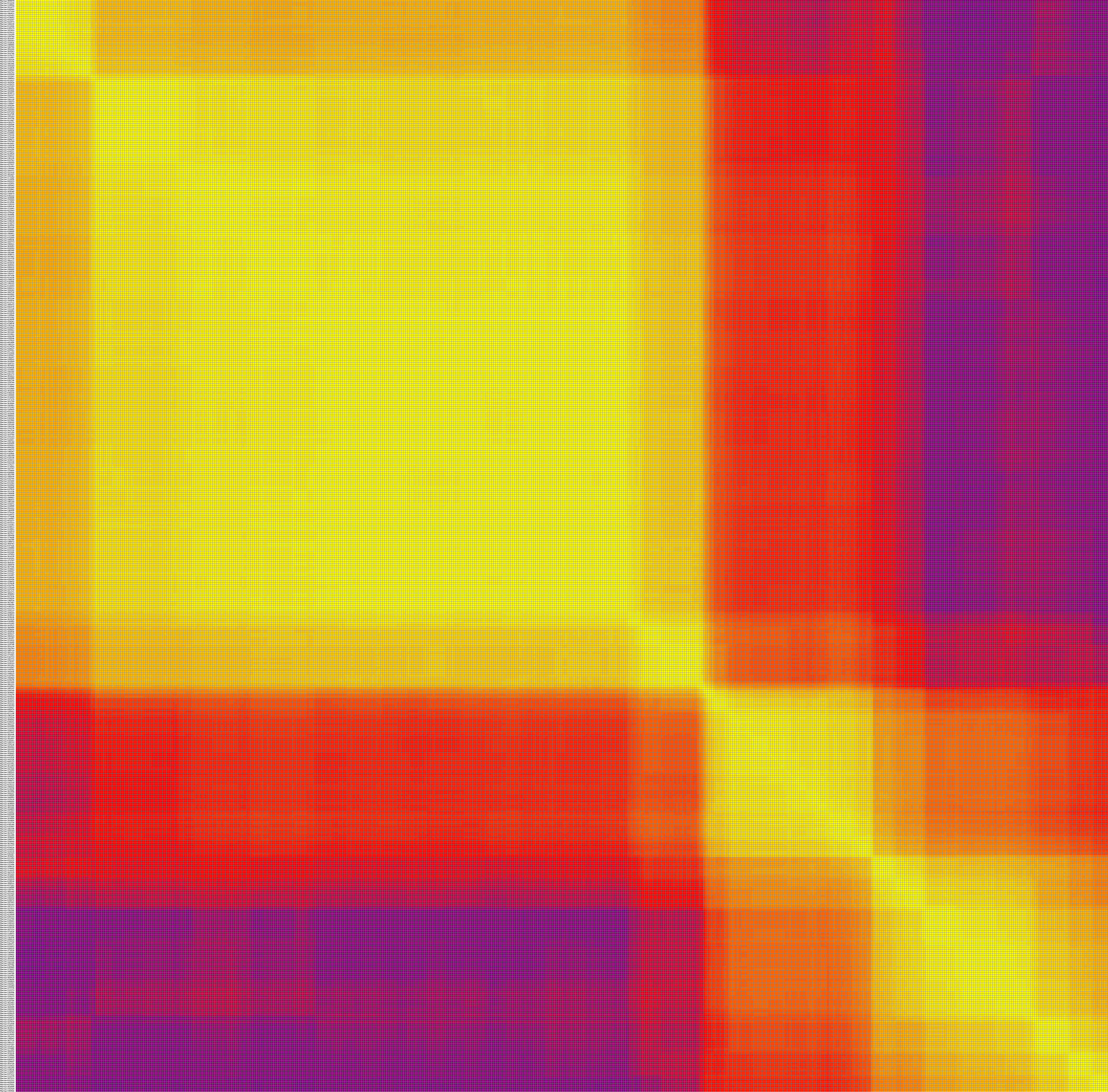

Supplement: FIGURE S4 — Haplotype map of the genetic map. Blue represents K1561, red represents G1025, green indicates heterozygous type, and gray represents deletions. [file Presentation_1.ZIP › Supplementary Figure S3/rice.Chr10.heatMap.png]

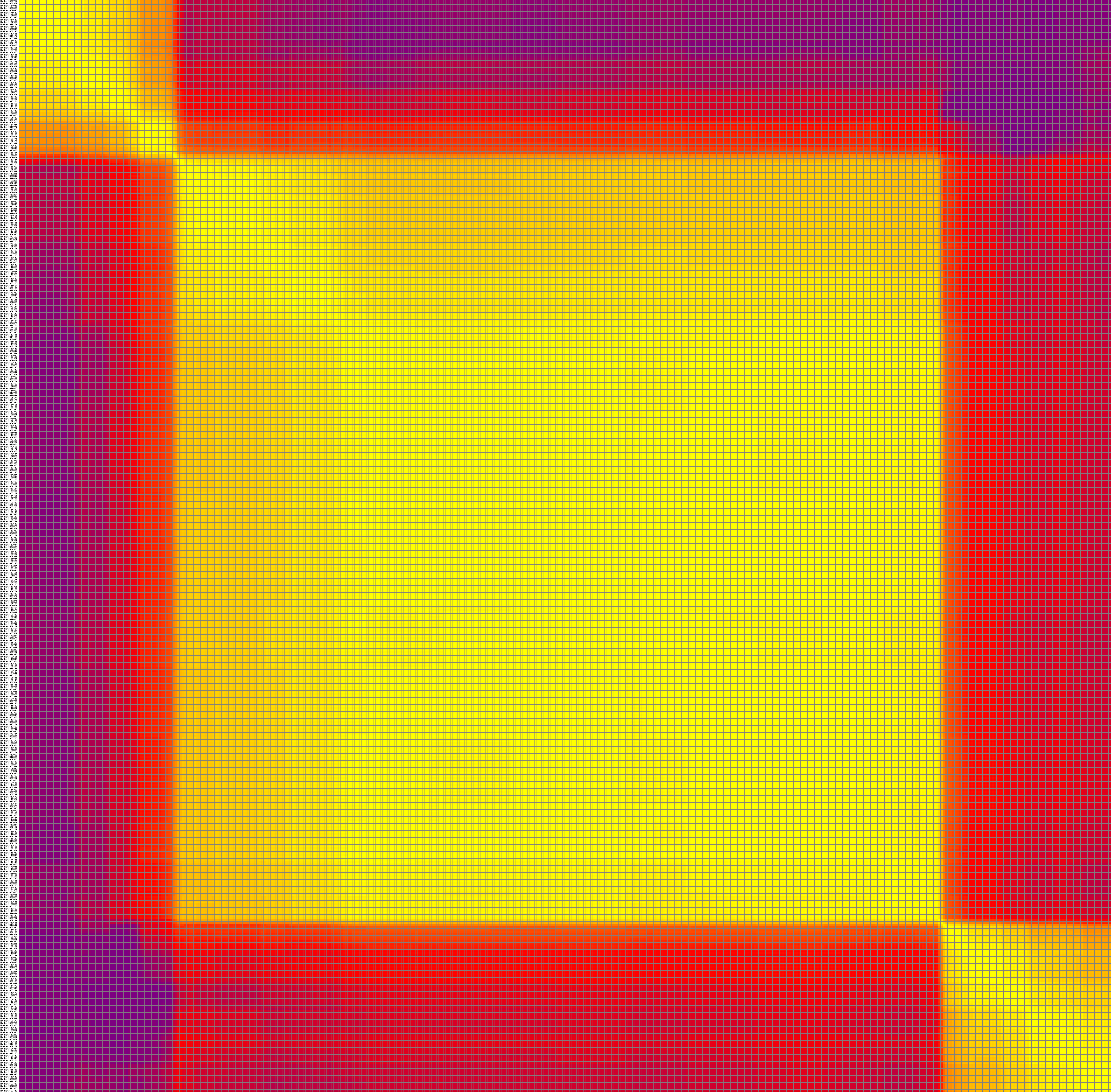

Supplement: FIGURE S4 — Haplotype map of the genetic map. Blue represents K1561, red represents G1025, green indicates heterozygous type, and gray represents deletions. [file Presentation_1.ZIP › Supplementary Figure S3/rice.Chr11.heatMap.png]

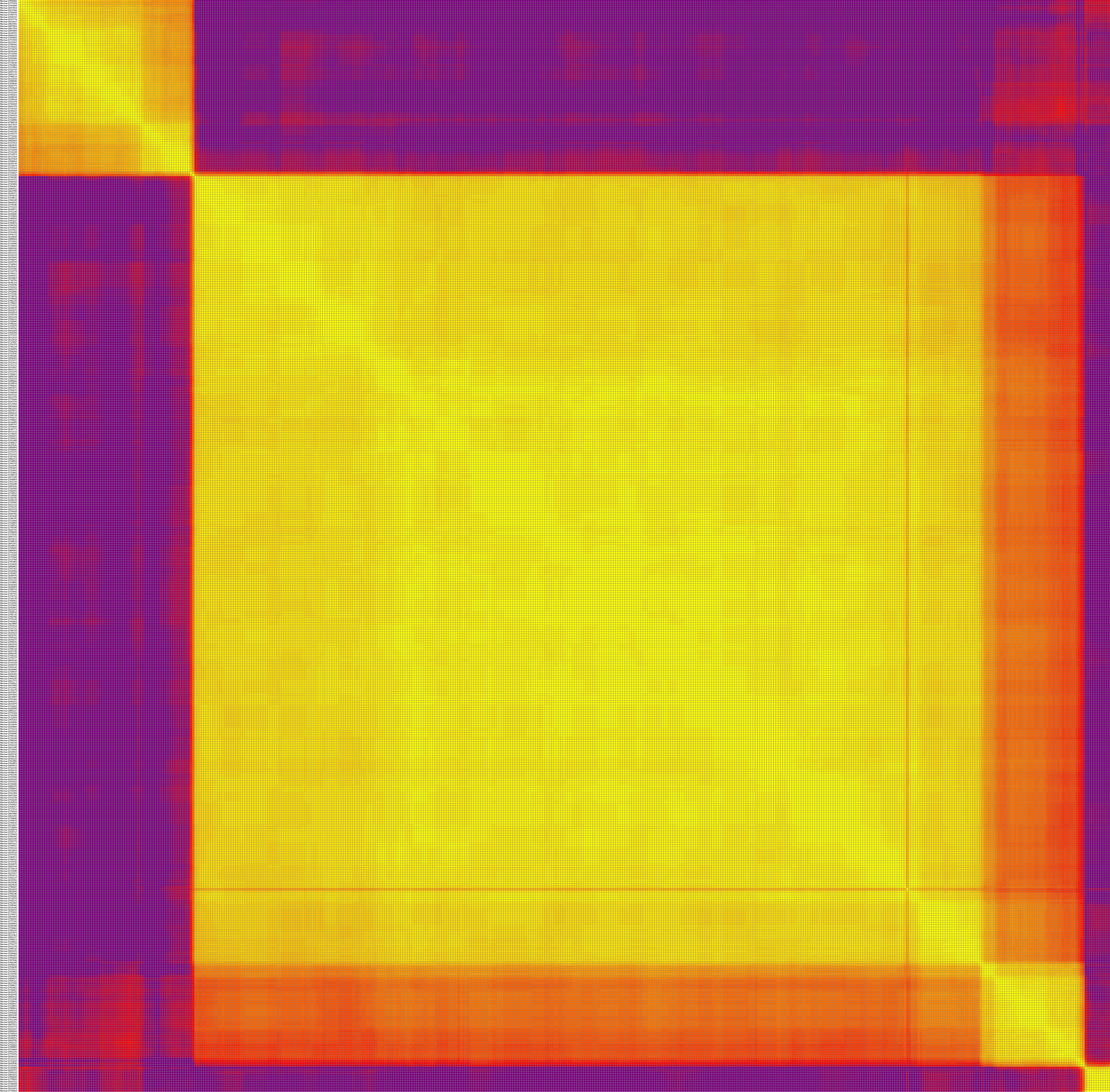

Supplement: FIGURE S4 — Haplotype map of the genetic map. Blue represents K1561, red represents G1025, green indicates heterozygous type, and gray represents deletions. [file Presentation_1.ZIP › Supplementary Figure S3/rice.Chr12.heatMap.png]

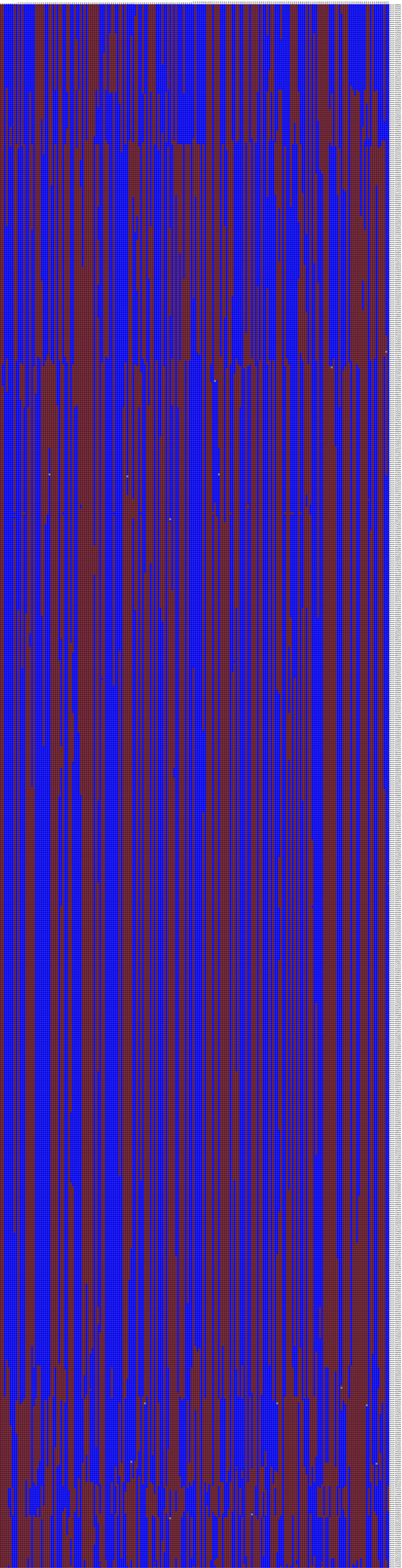

Supplement: FIGURE S5 — The collinearity of 12 chromosomes with the rice reference genome. The x-axis indicates the genetic distance of rice chromosomes, and the y-axis represents the linearity order of the physical position in the rice genome. All 5521 SLAF markers in these chromosomes are plotted as dots on the Figure. Different colors indicate different chromosomes. [file Presentation_2.ZIP › Supplementary Figure S4/rice.Chr01.haplo.png]

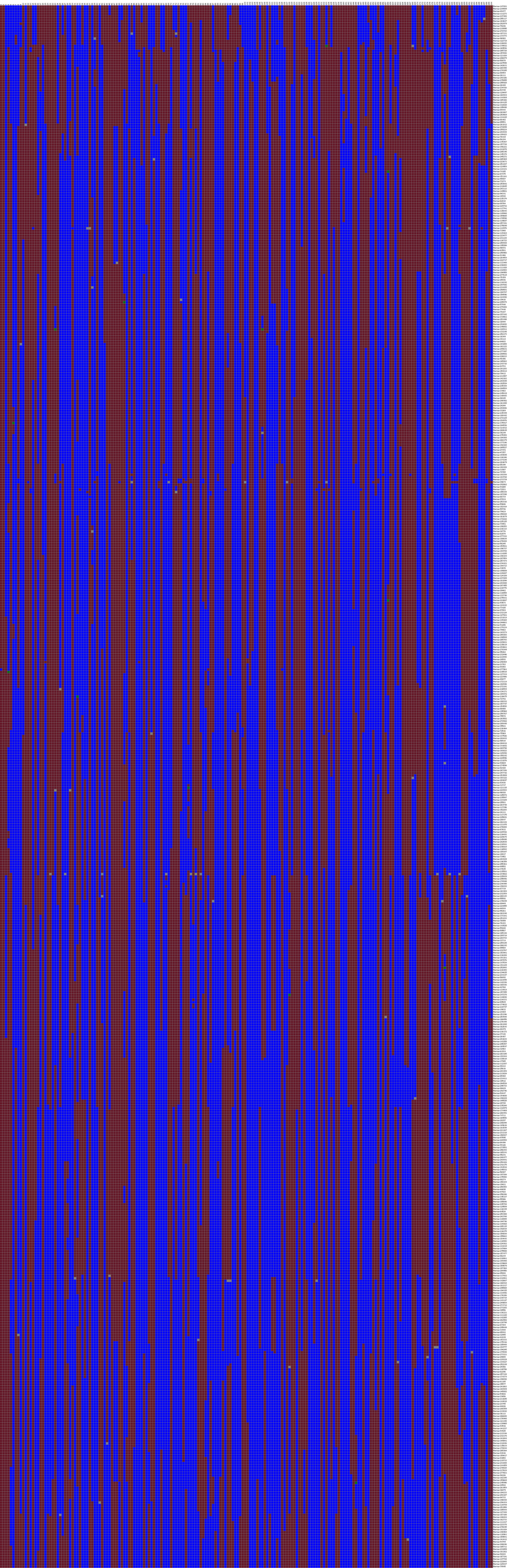

Supplement: FIGURE S5 — The collinearity of 12 chromosomes with the rice reference genome. The x-axis indicates the genetic distance of rice chromosomes, and the y-axis represents the linearity order of the physical position in the rice genome. All 5521 SLAF markers in these chromosomes are plotted as dots on the Figure. Different colors indicate different chromosomes. [file Presentation_2.ZIP › Supplementary Figure S4/rice.Chr02.haplo.png]

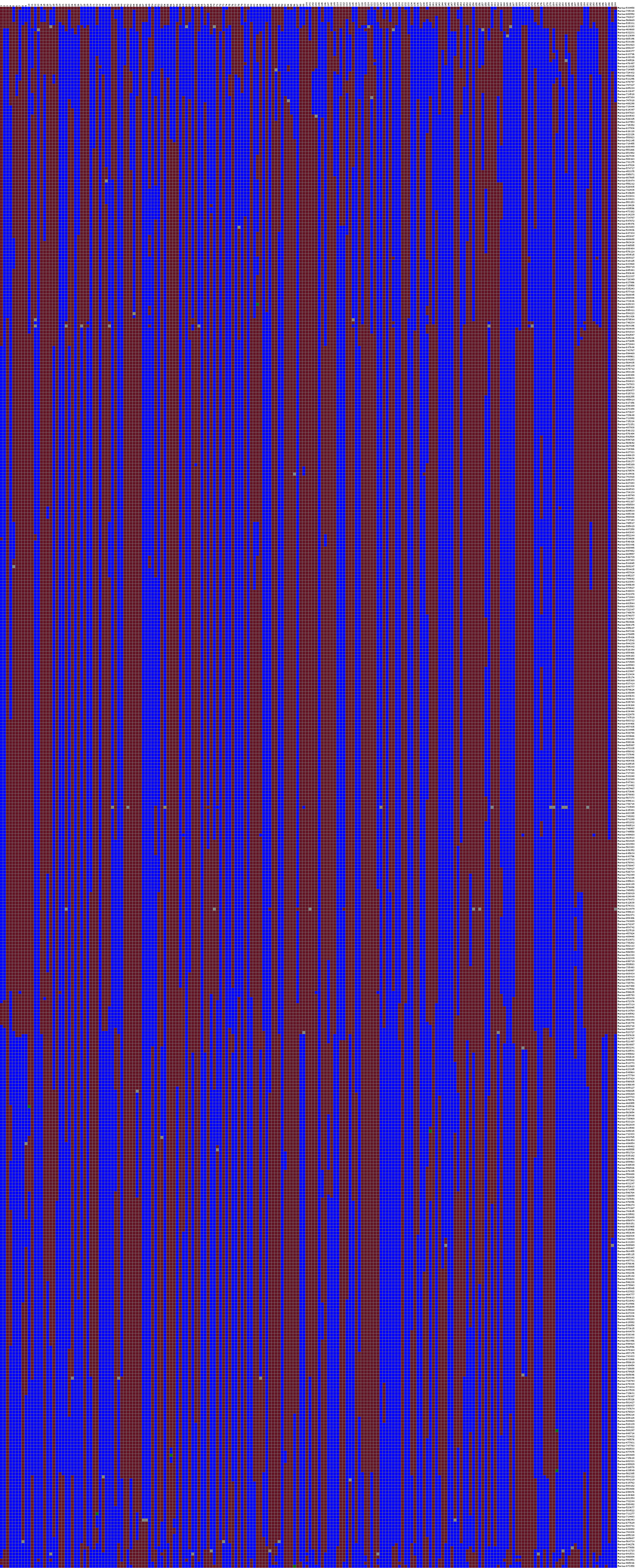

Supplement: FIGURE S5 — The collinearity of 12 chromosomes with the rice reference genome. The x-axis indicates the genetic distance of rice chromosomes, and the y-axis represents the linearity order of the physical position in the rice genome. All 5521 SLAF markers in these chromosomes are plotted as dots on the Figure. Different colors indicate different chromosomes. [file Presentation_2.ZIP › Supplementary Figure S4/rice.Chr03.haplo.png]

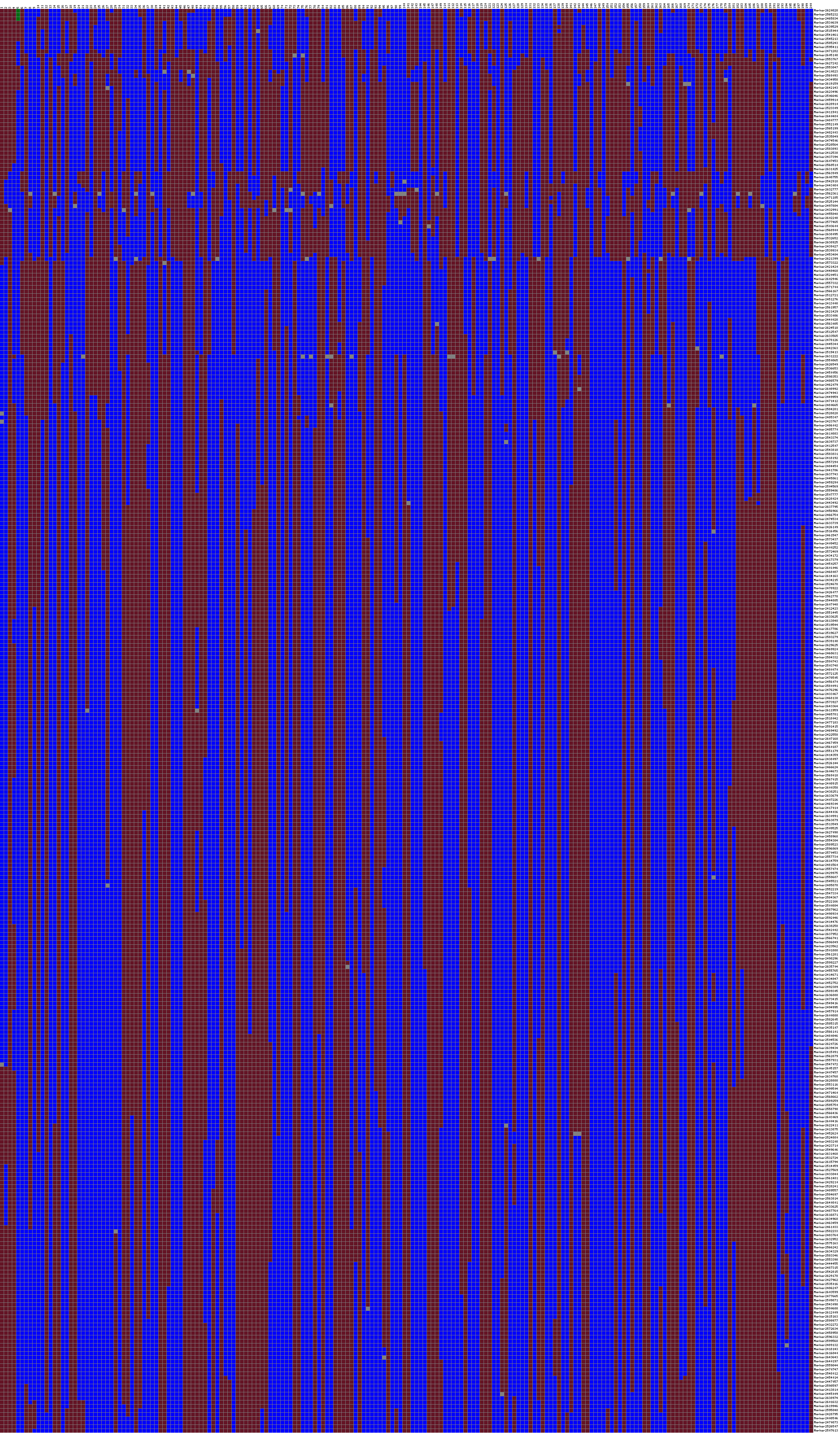

Supplement: FIGURE S5 — The collinearity of 12 chromosomes with the rice reference genome. The x-axis indicates the genetic distance of rice chromosomes, and the y-axis represents the linearity order of the physical position in the rice genome. All 5521 SLAF markers in these chromosomes are plotted as dots on the Figure. Different colors indicate different chromosomes. [file Presentation_2.ZIP › Supplementary Figure S4/rice.Chr04.haplo.png]

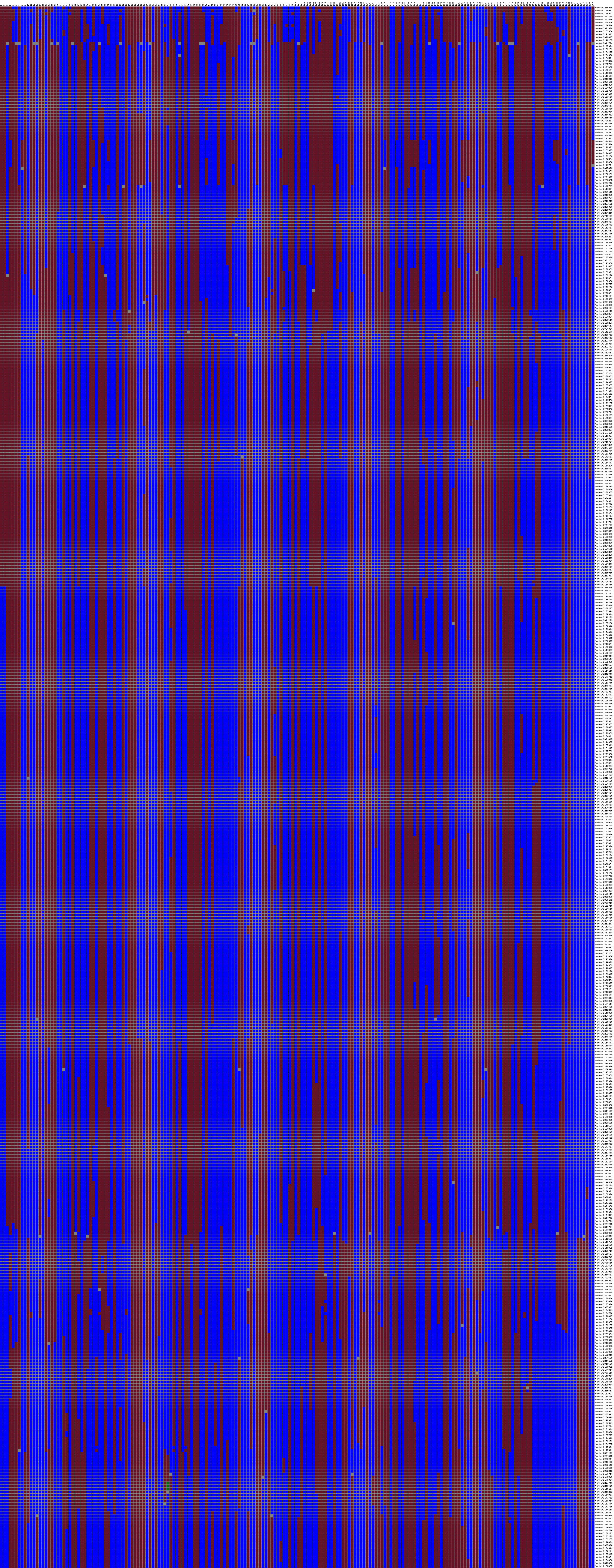

Supplement: FIGURE S5 — The collinearity of 12 chromosomes with the rice reference genome. The x-axis indicates the genetic distance of rice chromosomes, and the y-axis represents the linearity order of the physical position in the rice genome. All 5521 SLAF markers in these chromosomes are plotted as dots on the Figure. Different colors indicate different chromosomes. [file Presentation_2.ZIP › Supplementary Figure S4/rice.Chr05.haplo.png]

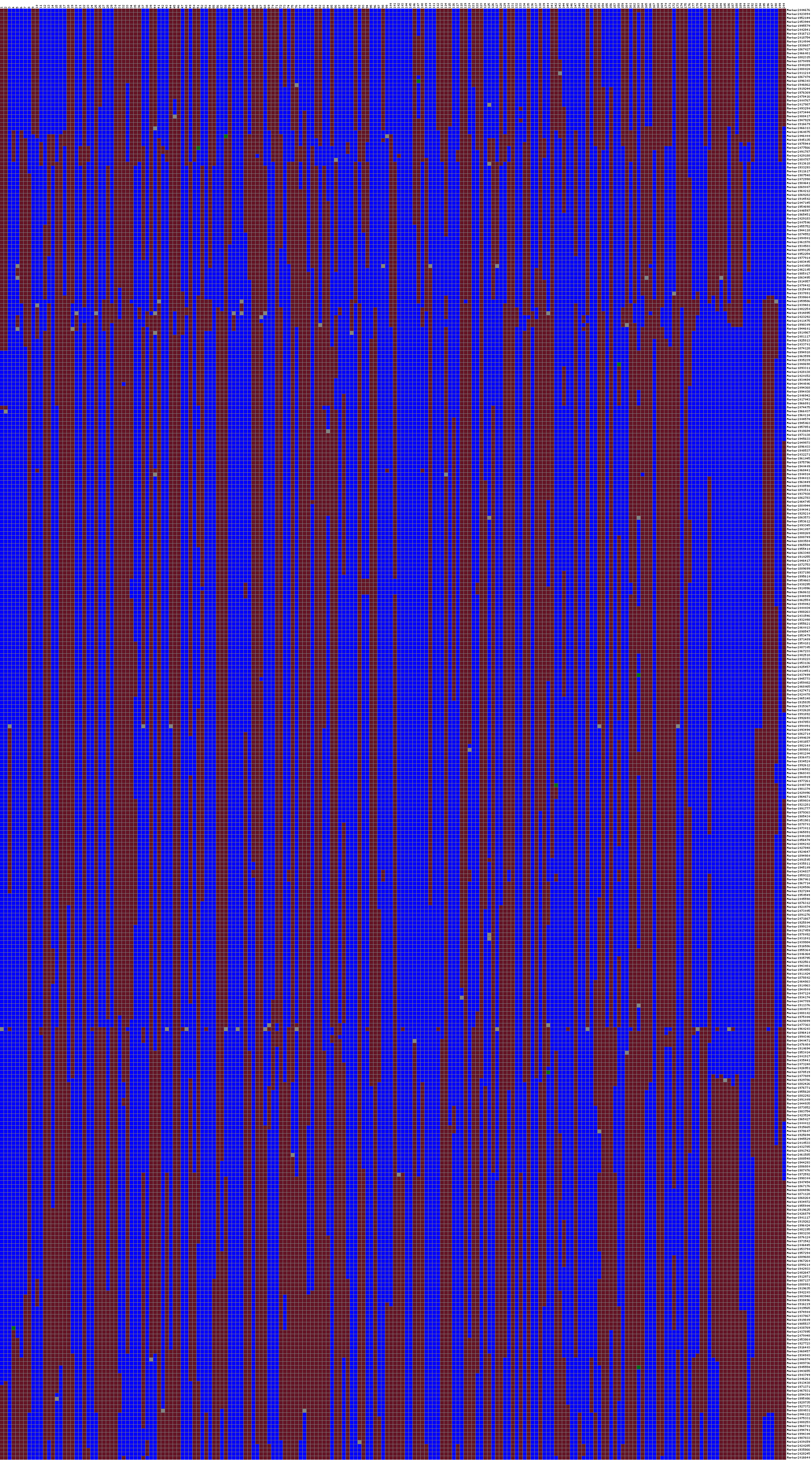

Supplement: FIGURE S5 — The collinearity of 12 chromosomes with the rice reference genome. The x-axis indicates the genetic distance of rice chromosomes, and the y-axis represents the linearity order of the physical position in the rice genome. All 5521 SLAF markers in these chromosomes are plotted as dots on the Figure. Different colors indicate different chromosomes. [file Presentation_2.ZIP › Supplementary Figure S4/rice.Chr06.haplo.png]

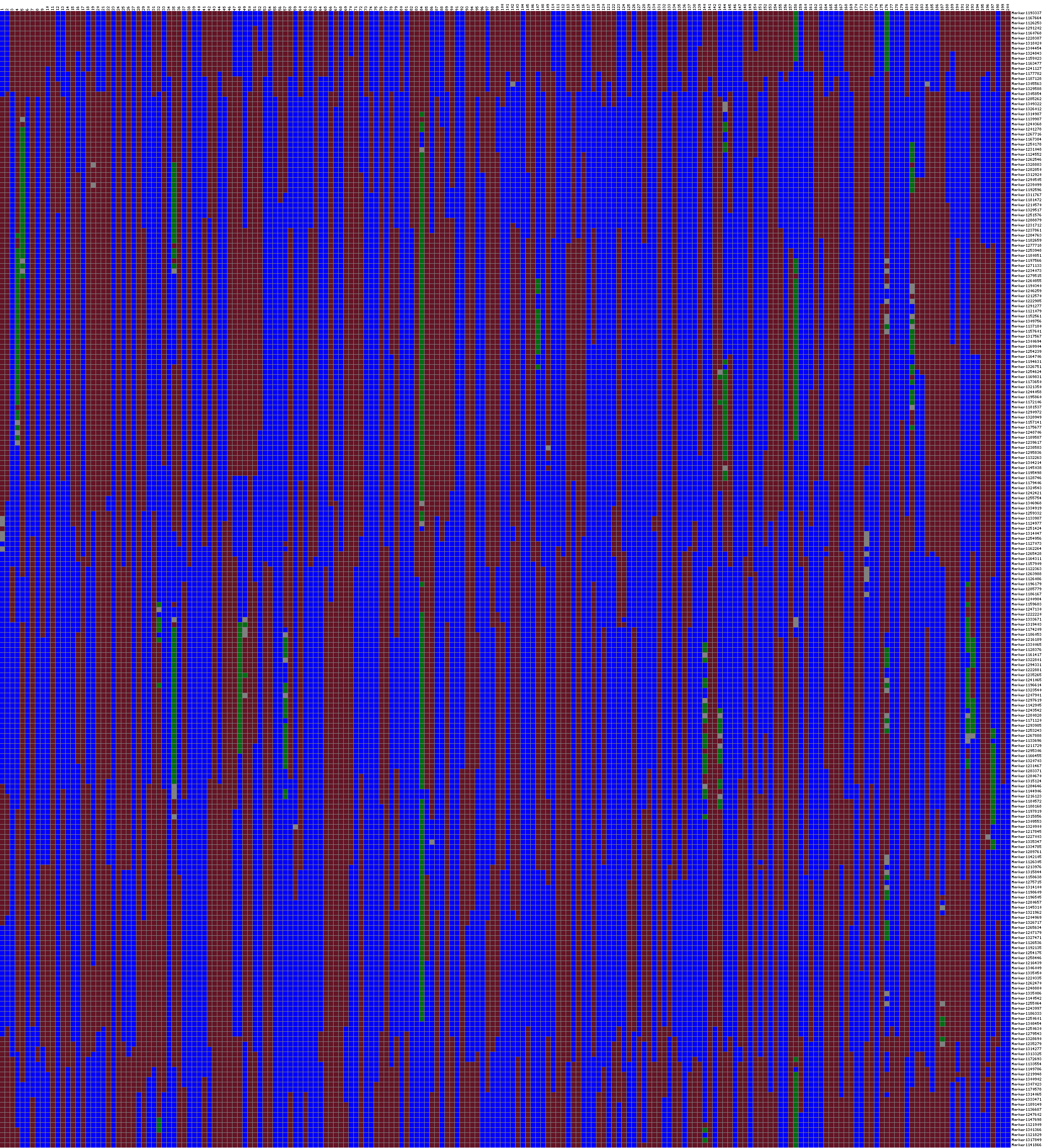

Supplement: FIGURE S5 — The collinearity of 12 chromosomes with the rice reference genome. The x-axis indicates the genetic distance of rice chromosomes, and the y-axis represents the linearity order of the physical position in the rice genome. All 5521 SLAF markers in these chromosomes are plotted as dots on the Figure. Different colors indicate different chromosomes. [file Presentation_2.ZIP › Supplementary Figure S4/rice.Chr07.haplo.png]

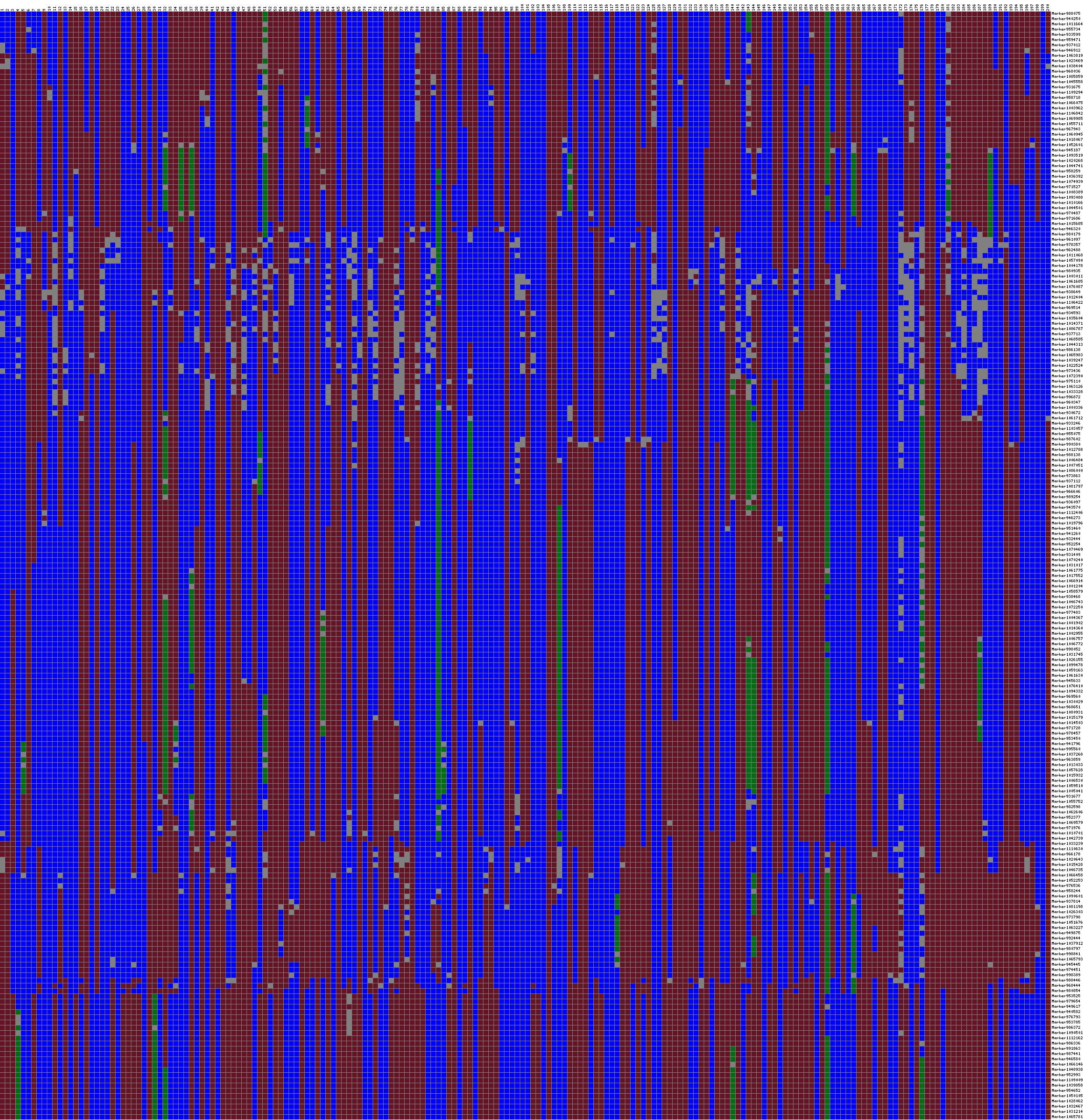

Supplement: FIGURE S5 — The collinearity of 12 chromosomes with the rice reference genome. The x-axis indicates the genetic distance of rice chromosomes, and the y-axis represents the linearity order of the physical position in the rice genome. All 5521 SLAF markers in these chromosomes are plotted as dots on the Figure. Different colors indicate different chromosomes. [file Presentation_2.ZIP › Supplementary Figure S4/rice.Chr08.haplo.png]

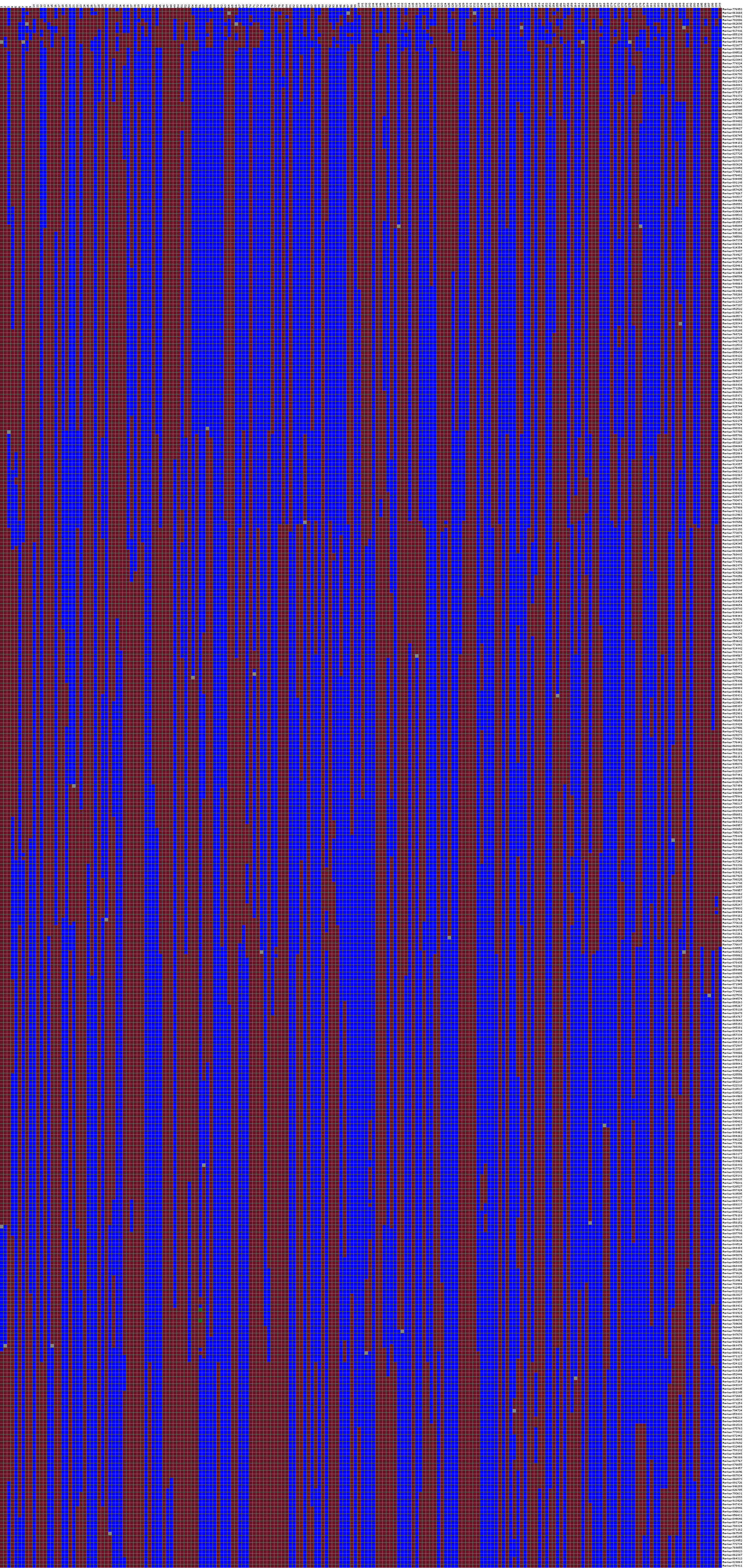

Supplement: FIGURE S5 — The collinearity of 12 chromosomes with the rice reference genome. The x-axis indicates the genetic distance of rice chromosomes, and the y-axis represents the linearity order of the physical position in the rice genome. All 5521 SLAF markers in these chromosomes are plotted as dots on the Figure. Different colors indicate different chromosomes. [file Presentation_2.ZIP › Supplementary Figure S4/rice.Chr09.haplo.png]

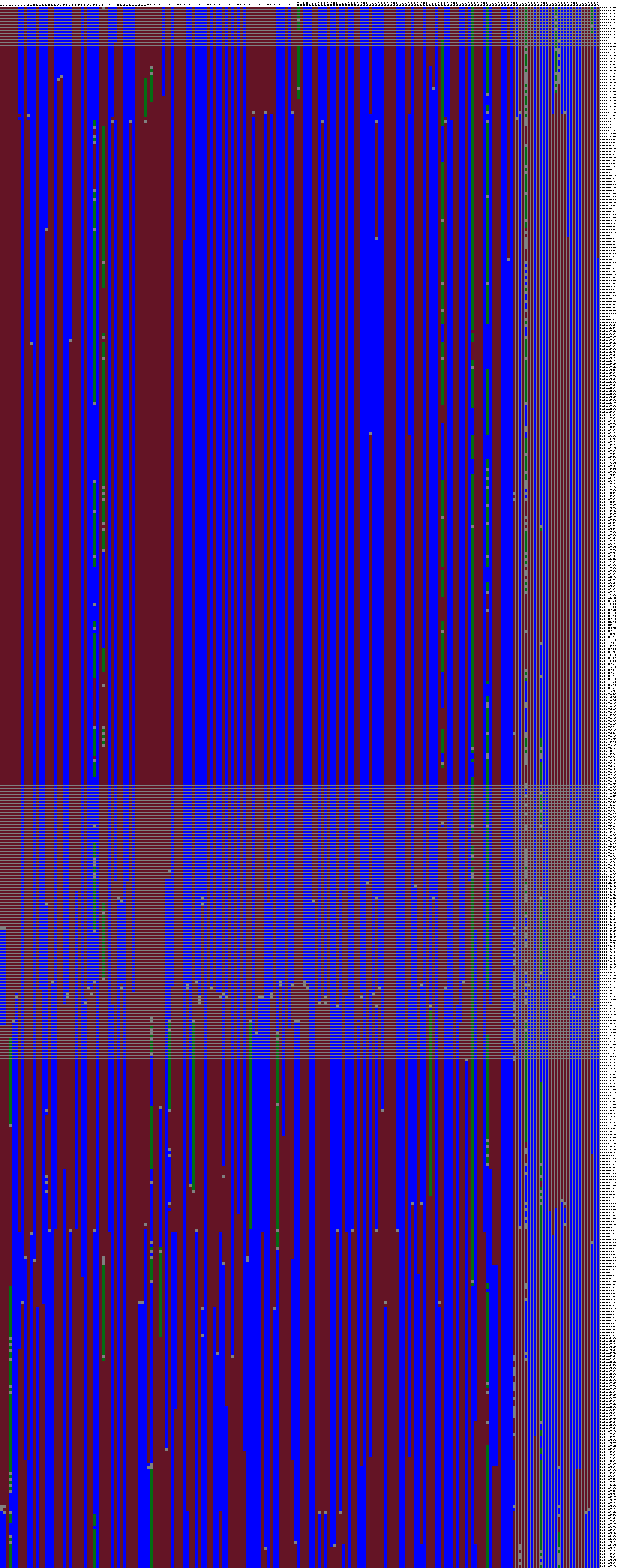

Supplement: FIGURE S5 — The collinearity of 12 chromosomes with the rice reference genome. The x-axis indicates the genetic distance of rice chromosomes, and the y-axis represents the linearity order of the physical position in the rice genome. All 5521 SLAF markers in these chromosomes are plotted as dots on the Figure. Different colors indicate different chromosomes. [file Presentation_2.ZIP › Supplementary Figure S4/rice.Chr10.haplo.png]

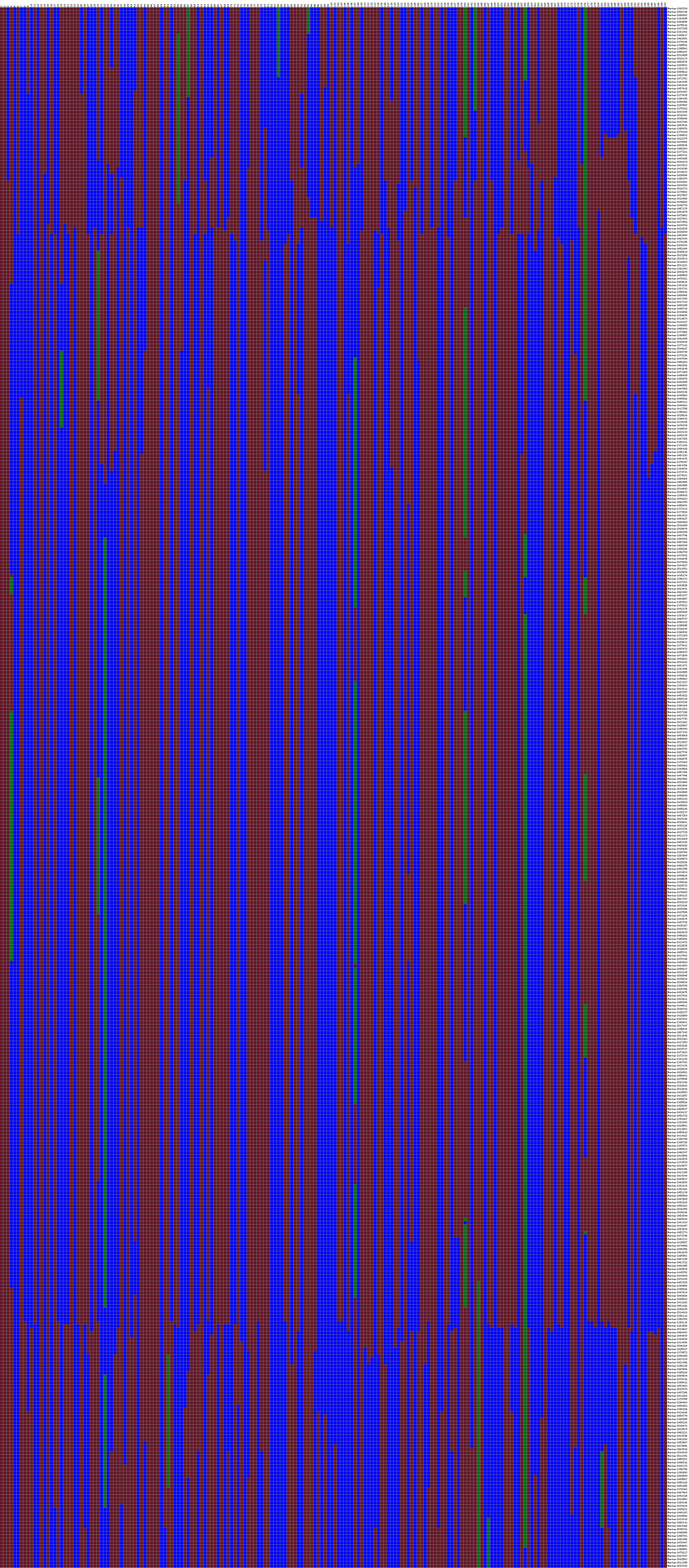

Supplement: FIGURE S5 — The collinearity of 12 chromosomes with the rice reference genome. The x-axis indicates the genetic distance of rice chromosomes, and the y-axis represents the linearity order of the physical position in the rice genome. All 5521 SLAF markers in these chromosomes are plotted as dots on the Figure. Different colors indicate different chromosomes. [file Presentation_2.ZIP › Supplementary Figure S4/rice.Chr11.haplo.png]

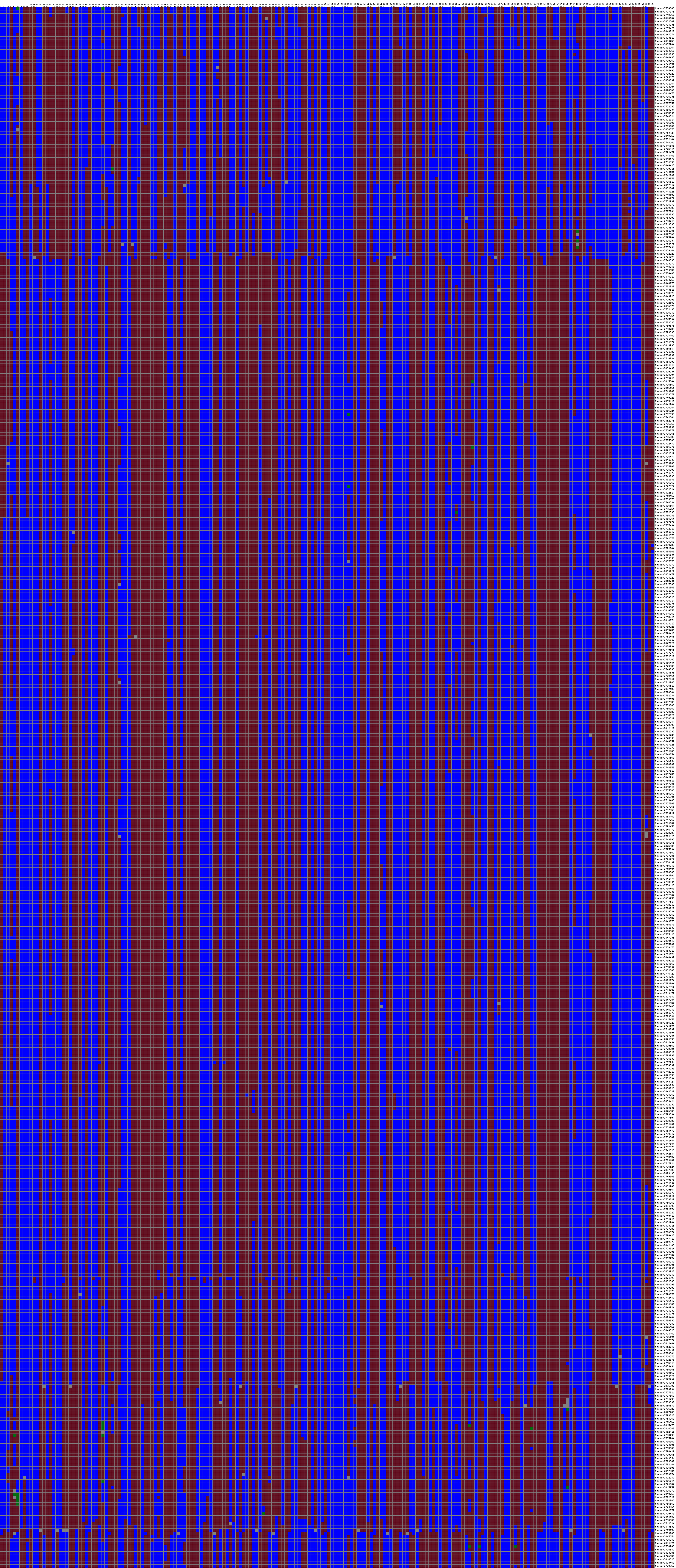

Supplement: FIGURE S5 — The collinearity of 12 chromosomes with the rice reference genome. The x-axis indicates the genetic distance of rice chromosomes, and the y-axis represents the linearity order of the physical position in the rice genome. All 5521 SLAF markers in these chromosomes are plotted as dots on the Figure. Different colors indicate different chromosomes. [file Presentation_2.ZIP › Supplementary Figure S4/rice.Chr12.haplo.png]
