# Supplementary material for: Identification of a Novel QTL for Panicle Length From Wild Rice (Oryza minuta) by Specific Locus Amplified Fragment Sequencing and High Density Genetic Mapping
Source: Front Plant Sci. 2018 Oct 16;9:1492. doi: 10.3389/fpls.2018.01492 (PMC6232755; doi:10.3389/fpls.2018.01492)
Supplement: TABLE S1 — Panicle length data of two parents and RILs. [file Table_1.DOCX]

**Supplementary Table S1 Panicle length data of two parents and RILs**

| RILs and parents | Plant location | | | |
| --- | --- | --- | --- | --- |
|  | 2014NN1  (cm) | 2014NN2  (cm) | 2015NN  (cm) | 2015WH  (cm) |
| GK1 | 21.02 | 21.22 | 23.08 | 22.24 |
| GK2 | 24.22 | 25.22 | 25.98 | 25.99 |
| GK3 | 21.96 | 25.96 | 23.53 | 24.01 |
| GK4 | 24.76 | 25.36 | 23.53 | 24.91 |
| GK5 | 27.03 | 27.57 | 28.30 | 27.87 |
| GK6 | 24.14 | 24.22 | 24.00 | 23.92 |
| GK7 | 24.16 | 25.16 | 25.18 | 25.74 |
| GK8 | 22.04 | 22.76 | 22.40 | 22.14 |
| GK9 | 22.58 | 22.98 | 25.40 | 24.52 |
| GK10 | 25.24 | 25.84 | 25.90 | 25.71 |
| GK11 | 21.48 | 22.88 | 24.43 | 23.61 |
| GK12 | 24.46 | 25.26 | 23.78 | 24.31 |
| GK13 | 18.44 | 21.24 | 20.70 | 20.25 |
| GK14 | 23.46 | 23.86 | 27.23 | 25.16 |
| GK15 | 22.28 | 23.68 | 24.65 | 23.59 |
| GK16 | 23.42 | 23.38 | 21.93 | 22.48 |
| GK17 | 21.11 | 21.09 | 25.75 | 23.26 |
| GK18 | 26.63 | 26.53 | 26.10 | 26.28 |
| GK19 | 24.12 | 23.92 | 24.53 | 24.45 |
| GK20 | 21.87 | 21.97 | 23.38 | 22.76 |
| GK21 | 24.08 | 23.88 | 28.05 | 26.17 |
| GK22 | 26.56 | 26.96 | 28.18 | 27.32 |
| GK23 | 26.66 | 26.86 | 28.48 | 27.94 |
| GK24 | 22.26 | 25.46 | 26.98 | 25.16 |
| GK25 | 24.46 | 24.26 | 25.33 | 24.68 |
| GK26 | 24.48 | 24.68 | 26.88 | 25.55 |
| GK27 | 25.76 | 27.76 | 26.53 | 26.96 |
| GK28 | 24.38 | 24.58 | 24.68 | 24.63 |
| GK29 | 22.72 | 23.12 | 27.58 | 25.20 |
| GK30 | 18.18 | 20.78 | 22.88 | 20.99 |
| GK31 | 23.88 | 24.08 | 28.78 | 26.40 |
| GK32 | 23.54 | 26.14 | 27.08 | 26.10 |
| GK33 | 26.22 | 26.18 | 25.60 | 25.73 |
| GK34 | 21.68 | 21.88 | 24.68 | 23.99 |
| GK35 | 19.33 | 19.19 | 22.00 | 20.76 |
| GK36 | 22.34 | 22.54 | 20.10 | 21.80 |
| GK37 | 23.00 | 22.96 | 23.10 | 22.71 |
| GK38 | 24.58 | 22.58 | 28.95 | 26.20 |
| GK39 | 23.16 | 22.96 | 25.23 | 24.06 |
| GK40 | 22.54 | 22.34 | 22.35 | 22.48 |
| GK41 | 25.34 | 25.46 | 26.78 | 25.61 |
| GK42 | 26.86 | 27.06 | 26.05 | 26.98 |
| GK44 | 21.18 | 23.18 | 26.75 | 24.62 |
| GK45 | 25.64 | 25.84 | 27.20 | 26.35 |
| GK46 | 24.78 | 24.86 | 26.88 | 26.34 |
| GK47 | 21.35 | 21.53 | 19.68 | 20.40 |
| GK48 | 22.69 | 22.75 | 23.20 | 23.14 |
| GK49 | 21.14 | 22.74 | 24.68 | 23.15 |
| GK50 | 23.56 | 23.60 | 25.35 | 24.94 |
| GK51 | 21.08 | 23.88 | 25.53 | 24.14 |
| GK52 | 22.55 | 22.49 | 27.08 | 24.54 |
| GK53 | 19.33 | 19.35 | 23.58 | 21.42 |
| GK54 | 24.22 | 24.14 | 30.53 | 27.51 |
| GK55 | 21.51 | 22.73 | 23.70 | 23.50 |
| GK56 | 22.22 | 22.10 | 25.67 | 23.66 |
| GK57 | 23.78 | 23.82 | 30.33 | 26.63 |
| GK58 | 24.98 | 24.58 | 28.85 | 26.84 |
| GK59 | 22.56 | 22.36 | 25.98 | 24.00 |
| GK60 | 23.78 | 23.82 | 28.03 | 25.48 |
| GK61 | 24.79 | 24.93 | 29.98 | 27.41 |
| GK62 | 23.67 | 25.81 | 25.13 | 25.49 |
| GK63 | 28.26 | 27.66 | 34.75 | 30.87 |
| GK64 | 22.82 | 22.62 | 23.68 | 23.62 |
| GK65 | 25.77 | 25.71 | 22.30 | 24.08 |
| GK66 | 22.10 | 21.94 | 24.13 | 23.01 |
| GK67 | 22.06 | 22.86 | 20.93 | 22.16 |
| GK68 | 23.88 | 23.80 | 27.20 | 25.64 |
| GK69 | 26.94 | 25.94 | 30.93 | 28.81 |
| GK70 | 27.32 | 29.16 | 27.20 | 27.89 |
| GK71 | 22.29 | 23.51 | 22.18 | 22.53 |
| GK72 | 21.55 | 21.53 | 20.08 | 21.56 |
| GK73 | 21.13 | 21.47 | 22.05 | 21.58 |
| GK74 | 20.55 | 20.37 | 24.28 | 22.27 |
| GK75 | 23.77 | 23.67 | 25.45 | 24.28 |
| GK76 | 24.99 | 24.89 | 26.45 | 26.00 |
| GK77 | 21.58 | 20.02 | 24.58 | 22.73 |
| GK78 | 17.67 | 17.73 | 19.10 | 18.29 |
| GK79 | 25.21 | 24.99 | 25.15 | 25.01 |
| GK80 | 19.66 | 19.54 | 20.93 | 20.15 |
| GK81 | 23.88 | 23.76 | 22.98 | 23.41 |
| GK82 | 24.55 | 24.41 | 27.23 | 25.52 |
| GK83 | 29.89 | 31.87 | 25.65 | 28.62 |
| GK84 | 26.44 | 26.32 | 23.45 | 24.87 |
| GK85 | 25.78 | 25.74 | 24.63 | 25.37 |
| GK86 | 25.32 | 25.12 | 26.60 | 24.93 |
| GK87 | 23.46 | 23.70 | 24.83 | 25.11 |
| GK88 | 25.84 | 24.24 | 27.78 | 26.30 |
| GK89 | 25.21 | 24.99 | 28.15 | 26.48 |
| GK90 | 24.89 | 24.83 | 29.45 | 26.48 |
| GK91 | 23.43 | 23.13 | 22.15 | 24.86 |
| GK92 | 26.88 | 26.08 | 27.23 | 24.47 |
| GK93 | 21.11 | 21.05 | 21.30 | 21.25 |
| GK94 | 20.55 | 20.37 | 20.80 | 21.28 |
| GK95 | 21.43 | 21.25 | 24.48 | 23.81 |
| GK96 | 23.44 | 23.40 | 25.88 | 24.12 |
| GK97 | 21.89 | 21.83 | 23.93 | 23.23 |
| GK98 | 22.44 | 22.36 | 25.65 | 24.56 |
| GK99 | 23.00 | 22.96 | 25.95 | 27.60 |
| GK100 | 28.93 | 30.13 | 30.73 | 29.30 |
| GK101 | 27.84 | 28.24 | 27.23 | 23.63 |
| GK102 | 19.66 | 19.46 | 24.33 | 24.89 |
| GK103 | 26.08 | 23.88 | 30.60 | 27.78 |
| GK104 | 24.01 | 26.19 | 30.33 | 29.19 |
| GK105 | 27.10 | 28.90 | 31.05 | 27.71 |
| GK106 | 24.86 | 23.46 | 26.33 | 24.42 |
| GK107 | 21.88 | 21.80 | 26.53 | 23.77 |
| GK108 | 21.66 | 21.58 | 21.73 | 23.87 |
| GK109 | 25.32 | 25.12 | 28.25 | 24.74 |
| GK110 | 21.66 | 21.58 | 29.30 | 26.36 |
| GK111 | 25.66 | 25.46 | 29.40 | 26.17 |
| GK112 | 22.76 | 22.48 | 26.60 | 24.35 |
| GK113 | 21.30 | 22.66 | 25.93 | 25.65 |
| GK114 | 24.77 | 24.67 | 26.43 | 25.83 |
| GK115 | 25.54 | 25.26 | 26.98 | 24.82 |
| GK116 | 22.08 | 23.28 | 24.18 | 24.43 |
| GK117 | 24.78 | 24.90 | 29.18 | 24.92 |
| GK118 | 20.57 | 20.75 | 22.35 | 24.81 |
| GK119 | 27.99 | 27.81 | 31.53 | 28.37 |
| GK120 | 24.78 | 24.82 | 23.08 | 26.26 |
| GK121 | 29.00 | 28.72 | 26.55 | 23.98 |
| GK122 | 20.77 | 20.71 | 24.83 | 24.51 |
| GK123 | 25.11 | 25.01 | 28.70 | 26.70 |
| GK124 | 24.21 | 23.99 | 28.45 | 28.47 |
| GK125 | 28.54 | 28.26 | 28.33 | 27.46 |
| GK126 | 26.43 | 26.17 | 26.28 | 28.75 |
| GK127 | 30.65 | 30.43 | 25.35 | 25.20 |
| GK128 | 25.32 | 25.12 | 23.13 | 23.08 |
| GK129 | 23.55 | 23.41 | 24.28 | 22.52 |
| GK130 | 20.89 | 20.75 | 22.48 | 22.21 |
| GK131 | 22.33 | 22.27 | 24.88 | 22.32 |
| GK132 | 20.35 | 20.25 | 17.63 | 19.85 |
| GK133 | 20.38 | 20.18 | 24.45 | 24.73 |
| GK134 | 25.58 | 25.42 | 27.88 | 26.39 |
| GK135 | 25.12 | 25.72 | 24.65 | 25.53 |
| GK136 | 26.22 | 26.18 | 29.70 | 27.72 |
| GK137 | 25.43 | 25.25 | 29.00 | 27.34 |
| GK138 | 26.00 | 24.84 | 25.50 | 25.05 |
| GK139 | 24.79 | 24.97 | 24.65 | 23.73 |
| GK140 | 21.34 | 21.38 | 24.03 | 24.22 |
| GK141 | 24.53 | 24.15 | 30.05 | 26.52 |
| GK142 | 23.32 | 23.04 | 22.93 | 25.63 |
| GK143 | 26.78 | 28.82 | 28.18 | 26.93 |
| GK144 | 26.01 | 26.03 | 23.95 | 22.66 |
| GK145 | 20.18 | 20.38 | 22.83 | 22.28 |
| GK146 | 20.78 | 20.98 | 23.15 | 19.65 |
| GK147 | 16.11 | 16.25 | 31.38 | 25.73 |
| GK148 | 21.26 | 21.46 | 20.85 | 22.40 |
| GK149 | 23.64 | 23.44 | 27.08 | 25.84 |
| GK150 | 24.01 | 24.19 | 30.48 | 26.64 |
| GK151 | 23.00 | 23.12 | 25.93 | 25.27 |
| GK152 | 24.66 | 24.58 | 27.48 | 27.94 |
| GK153 | 27.33 | 27.31 | 27.33 | 26.40 |
| GK154 | 25.24 | 25.64 | 28.55 | 25.49 |
| GK155 | 22.10 | 21.94 | 22.58 | 23.98 |
| GK156 | 25.81 | 24.47 | 29.20 | 25.76 |
| GK157 | 22.55 | 22.37 | 23.70 | 22.73 |
| GK158 | 21.66 | 21.58 | 22.75 | 23.66 |
| GK159 | 24.66 | 24.62 | 28.30 | 26.94 |
| GK160 | 25.82 | 25.82 | 28.38 | 26.67 |
| GK161 | 24.68 | 24.68 | 25.10 | 25.09 |
| GK162 | 25.04 | 24.88 | 28.13 | 27.26 |
| GK163 | 27.43 | 26.97 | 26.15 | 25.19 |
| GK164 | 24.23 | 22.85 | 23.53 | 24.99 |
| GK165 | 26.55 | 26.37 | 26.60 | 27.76 |
| GK166 | 26.54 | 28.26 | 25.65 | 27.04 |
| GK167 | 26.55 | 28.45 | 26.93 | 26.33 |
| GK168 | 25.78 | 25.86 | 29.38 | 26.71 |
| GK169 | 24.12 | 23.92 | 26.33 | 24.67 |
| GK170 | 23.25 | 23.07 | 25.15 | 25.50 |
| GK171 | 25.28 | 25.20 | 26.18 | 25.56 |
| GK172 | 25.25 | 24.75 | 26.88 | 24.73 |
| GK173 | 22.18 | 23.42 | 23.25 | 23.72 |
| GK174 | 24.12 | 24.24 | 26.00 | 26.63 |
| GK175 | 27.32 | 27.16 | 27.28 | 26.08 |
| GK176 | 23.08 | 25.28 | 25.68 | 24.71 |
| GK177 | 23.56 | 23.76 | 26.03 | 27.37 |
| GK178 | 28.98 | 30.78 | 28.48 | 29.45 |
| GK179 | 28.88 | 30.76 | 27.95 | 27.90 |
| GK180 | 27.34 | 27.46 | 34.20 | 29.22 |
| GK181 | 24.77 | 24.63 | 29.23 | 28.06 |
| GK182 | 26.32 | 25.92 | 24.28 | 25.82 |
| GK183 | 27.18 | 26.98 | 24.68 | 25.49 |
| GK184 | 25.28 | 25.08 | 26.03 | 25.80 |
| GK185 | 25.78 | 25.82 | 24.33 | 24.39 |
| GK186 | 23.89 | 23.95 | 24.98 | 24.57 |
| GK187 | 24.77 | 24.71 | 24.98 | 24.93 |
| GK188 | 24.88 | 24.64 | 25.38 | 25.12 |
| GK189 | 24.34 | 24.14 | 24.28 | 23.98 |
| GK190 | 23.54 | 23.26 | 23.08 | 24.70 |
| GK191 | 25.77 | 25.59 | 24.63 | 25.62 |
| GK192 | 27.14 | 26.94 | 25.75 | 25.77 |
| GK193 | 26.65 | 28.39 | 33.58 | 30.33 |
| GK194 | 25.34 | 25.38 | 25.95 | 25.96 |
| GK195 | 23.89 | 23.83 | 28.73 | 26.20 |
| GK196 | 25.42 | 23.82 | 25.38 | 25.30 |
| GK197 | 24.32 | 21.72 | 27.30 | 25.17 |
| GK198 | 25.77 | 25.71 | 26.60 | 26.41 |
| GK199 | 23.32 | 23.16 | 25.70 | 24.46 |
| GK200 | 23.12 | 22.84 | 24.98 | 24.37 |
| GK201 | 26.44 | 28.34 | 28.00 | 27.72 |
| K1561 | 29.32 | 30.92 | 30.28 | 30.29 |
| G1025 | 20.52 | 20.68 | 20.80 | 21.23 |
